# Divergent Peptide Presentations of HLA-A^*^30 Alleles Revealed by Structures With Pathogen Peptides

**DOI:** 10.3389/fimmu.2019.01709

**Published:** 2019-07-23

**Authors:** Shiyan Zhu, Kefang Liu, Yan Chai, Yanan Wu, Dan Lu, Wenling Xiao, Hao Cheng, Yingze Zhao, Chunming Ding, Jianxin Lyu, Yongliang Lou, George F. Gao, William J. Liu

**Affiliations:** ^1^School of Laboratory Medicine and Life Sciences, Wenzhou Medical University, Wenzhou, China; ^2^NHC Key Laboratory of Medical Virology and Viral Diseases, Chinese Center for Disease Control and Prevention, National Institute for Viral Disease Control and Prevention, Beijing, China; ^3^Faculty of Health Sciences, University of Macau, Macau, China; ^4^CAS Key Laboratory of Pathogenic Microbiology and Immunology, Institute of Microbiology, Chinese Academy of Sciences, Beijing, China; ^5^Beijing Institutes of Life Science, University of Chinese Academy of Sciences, Beijing, China; ^6^Hangzhou Medical College, Hangzhou, China

**Keywords:** HLA-A3, HLA superfamily, cross-presentation, influenza virus, human immunodeficiency virus (HIV), *Mycobacterium tuberculosis*, T-cell, major histocompatibility complex (MHC)

## Abstract

Human leukocyte antigen (HLA) alleles have a high degree of polymorphism, which determines their peptide-binding motifs and subsequent T-cell receptor recognition. The simplest way to understand the cross-presentation of peptides by different alleles is to classify these alleles into supertypes. A1 and A3 HLA supertypes are widely distributed in humans. However, direct structural and functional evidence for peptide presentation features of key alleles (e.g., HLA-A^*^30:01 and -A^*^30:03) are lacking. Herein, the molecular basis of peptide presentation of HLA-A^*^30:01 and -A^*^30:03 was demonstrated by crystal structure determination and thermostability measurements of complexes with T-cell epitopes from influenza virus (NP44), human immunodeficiency virus (RT313), and *Mycobacterium tuberculosis* (MTB). When binding to the HIV peptide, RT313, the PΩ-Lys anchoring modes of HLA-A^*^30:01, and -A^*^30:03 were similar to those of HLA-A^*^11:01 in the A3 supertype. However, HLA-A^*^30:03, but not -A^*^30:01, also showed binding with the HLA^*^01:01-favored peptide, NP44, but with a specific structural conformation. Thus, different from our previous understanding, HLA-A^*^30:01 and -A^*^30:03 have specific peptide-binding characteristics that may lead to their distinct supertype-featured binding peptide motifs. Moreover, we also found that residue 77 in the F pocket was one of the key residues for the divergent peptide presentation characteristics of HLA-A^*^30:01 and -A^*^30:03. Interchanging residue 77 between HLA-A^*^30:01 and HLA-A^*^30:03 switched their presented peptide profiles. Our results provide important recommendations for screening virus and tumor-specific peptides among the population with prevalent HLA supertypes for vaccine development and immune interventions.

## Introduction

The major histocompatibility complex (MHC), also known as the human leukocyte antigen (HLA) system in humans, plays a pivotal role in activating T-cell immune responses by presenting antigen peptides on antigen-presenting cells (APCs). The residues in the peptide-binding grooves (PBGs) of HLA molecules are highly polymorphic and this determines the peptide-binding motifs and the subsequent T-cell immunity features of people with different HLA types. At the present moment, 15,586 HLA molecules have been identified (https://www.ebi.ac.uk/ipd/imgt/hla/stats.html). The concept of HLA supertypes offers a simple and useful way to clarify the peptide-binding features of HLA molecules with cross-presented peptide reservoirs (1–3). In particular, determining the MHC-binding peptide motif is now widely accessible as an important approach of MHC I allele classification (4). Twelve HLA supertypes (A1, A1A3, A1A24, A2, A3, A24, B7, B8, B27, B44, B58, and B62) have thus far been reported, based on their binding motifs (4–8). Alleles of the same supertype have similar binding motifs, which is very useful for screening special peptides presented by certain MHC I molecules and would benefit subsequent T-cell recognition and vaccine development studies.

The A^*^30 serotype, initially identified as a split of a serologically cross-reactive group of antigens known as HLA-A19 (9), is one of the key serotypes among the population, and can be further subdivided, using isoelectric focusing or primed cytotoxic T-cell lines (CTL), into A^*^30:01, A^*^30:02, A^*^30:03, etc., which are genetically related (10–12). However, based on the current definition, alleles from the A^*^30 serotype possess different peptide-binding motifs and belong to distinct HLA supertypes including A1 and A3, both of which are widely distributed in humans (4). According to previous studies based on binding assays or pool sequencing/ligand elution, the A1 supertype includes HLA-A^*^01:01, HLA-A^*^26:01, HLA-A^*^30:03, HLA-A^*^30:04, and HLA-A^*^32:01. A1 supertype alleles prefer to have amino acids which are either small or aliphatic (e.g., Ala, Thr, Ser, Val, Leu, Ile, Met, or Gln) at position 2 (P2) of the peptide and aromatic and large hydrophobic amino acids (e.g., Phe, Trp, Tyr, Leu, Ile, and Met) at the C-terminus (PΩ) (4). The A3 supertype includes HLA-A^*^03:01, HLA-A^*^11:01, HLA-A^*^31:01, HLA-A^*^33:01, HLA-A^*^66:01, and HLA-A^*^68:01. A3 supertype alleles also prefer amino acids which are either small or aliphatic at P2 of the peptides they present. However, basic amino acids (e.g., Arg, His, and Lys) are favored as the PΩ residue of peptides presented by A3 supertype molecules (4, 13).

HLA-A^*^30:01 and HLA-A^*^30:03 differ from one another only by five amino acids, three of which are located in the PBG (14). However, the binding motifs of these two HLA alleles are quite different from each other (4). Thus far, the supertype classification of HLA-A^*^30:01 and HLA-A^*^30:03 is still in debate. Initially, HLA-A^*^30:01 was classified as an A24 supertype based on sequence and biological data (5), but was later reclassified as an A1 supertype using clustering of specificity matrices (6). To further complicate matters, HLA-A^*^30:01 was also assigned to the A3 supertype according to bioinformatic methods (15) and then later reassigned to the A3 supertype, based on HLA-A^*^30:01 binding assays (16). Meanwhile, others proposed that HLA-A^*^30:01 belonged to the A1A3 supertype, based on a compilation of published motifs, binding data, and analyses of shared repertoires of binding peptides (4). In the same study, HLA-A^*^30:03 was reported to be an A1 supertype allele (4). Clearly, the molecular bases of peptide presentation by HLA-A^*^30:01 and HLA-A^*^30:03 still need to be determined in order to precisely classify these two closely related HLA alleles.

In this study, we determined the peptide presentation features of HLA-A^*^30:01 and HLA-A^*^30:03 through a series of structural and functional investigations. In contrast to what was previously reported, we found that HLA-A^*^30:01 and HLA-A^*^30:03 may have the peptide-binding features of the A3 and A1A3 supertype, respectively. Interestingly, we found that residue 77 is key for determining the different binding motifs between HLA-A^*^30:01 and HLA-A^*^30:03. Our results increase the understanding of HLA supertypes and pave the way for peptide screening and vaccine development based on the binding motifs of different supertypes.

## Materials and Methods

### Peptide Synthesis

The peptide, NP44 (CTELKLSDY) derived from the influenza virus nucleocapsid protein (residues 44–52), RT313 (AIFQSSMTK) from the HIV reverse transcriptase (residues 313–321), and MTB (QIMYNYPAM) from *Mycobacterium tuberculosis* protein TB10.4 were synthesized and purified to 90% by reverse-phase HPLC and mass spectrometry (SciLight Biotechnology, Beijing, China) (Table 1). These peptides were stored in lyophilized aliquots at −80°C after synthesis and dissolved in dimethyl sulfoxide (DMSO) before use. Other peptides used in this paper were synthesized and purified in the same way (Tables 2, 3).

**Table 1:** Characteristics of the peptides used in structure determinations.

**Name**	**Derived protein**	**Position**	**Sequence^a^**	**Refolding^b^**		**References**
				A*30:01	A*30:03	
NP44	Influenza virus NP protein	44–52	C**T**ELKLSD**Y**	–	+	(17)
MTB	MTB^c^ protein TB10.4	3–11	Q**I**MYNYPA**M**	–	+	(18)
RT313	HIV RT protein	313–321	A**I**FQSSMT**K**	+	+	(13, 19)

a Underlined boldface residues are the typical primary anchors of the peptides presented by HLA-A^*^30:01 or -A*30:03.

b Peptides that can help the HLA-A^*^30:01 or -A*30:03 H chain renature with human β_2_ microglobulin (β_2_m) are marked as +, otherwise as –.

c *MTB, Mycobacterium tuberculosis*.

**Table 2:** Binding assays of HLA-A^*^30:01 or -A*30:03 with peptide MTB and its mutants.

**Name**	**Sequence^a^**	**Refolding^b^**	
		A*30:01	A*30:03
MTB	QIMYNYPA**M**	–	+
MTB-M9K	QIMYNYPA**K**	+	+
MTB-M9R	QIMYNYPA**R**	+	–
MTB-M9Y	QIMYNYPA**Y**	–	+
MTB-M9F	QIMYNYPA**F**	–	+
MTB-M9I	QIMYNYPA**I**	–	+
MTB-M9V	QIMYNYPA**V**	–	+
MTB-M9L	QIMYNYPA**L**	–	+
MTB-M9T	QIMYNYPA**T**	–	+
MTB-M9S	QIMYNYPA**S**	–	+

a *Underlined boldface residues are the substituted sites*.

b In the in vitro refolding assay, peptides that can help the HLA-A^*^30:01 or -A*30:03 H chain renature with human β_2_ microglobulin (β_2_m) are marked as +, otherwise as –.

**Table 3:** Binding assays of HLA-A^*^30:01 or -A*30:03 with published peptides which were reported to be presented by HLA-A^*^3001 in the IEDB.

**Name**	**Sequence**	**Refolding^a^**		**References**
		A*30:01	A*30:03	
IEDB1	IMYNYPAML	–	+	(18)
IEDB2	LVRAYHAMS	–	+	(18)
IEDB3	AAYHPQQFI	–	–	(10)
IEDB4	DGRDGGICIFN	–	–	(20)
IEDB5	DSDSSNPALQV	–	–	(20)
IEDB6	EIFGLHENV	–	–	(20)
IEDB7	ELKLRGLPVSGT	–	–	(20)
IEDB8	VLDTPGPPV	–	–	(20)
IEDB9	LYKDVMQETI	–	+	(20)
IEDB10	PTEQPQAWAV	–	–	(20)
IEDB11	QSMFTCKTEV	–	+	(20)
IEDB12	SPRPSVPAP	–	–	(20)
IEDB13	VLDLVDPV	–	–	(20)
IEDB14	LGRVRDGP	–	–	(20)
IEDB15	LAKLPMPKIHY	–	+	(20)

a Peptides that can help the HLA-A^*^30:01 or -A*30:03 H chain renature with human β_2_ microglobulin (β_2_m) are marked as +, otherwise as –.

### Plasmids

The extracellular regions (residues 1–274) of the MHC class I heavy (H) chain, HLA-A^*^30:01 (GenBank accession no. ACA34998.1), and HLA-A^*^30:03 (GenBank accession no. ANG08799.1) were synthesized (Genewiz, Suzhou, China) and cloned into pET21a (+) vectors. The expression plasmid for human β_2_ microglobulin (β_2_m, expressing residues 1–99) was constructed in our laboratory (21). HLA-A^*^30:01D77N (with residue 77 mutated from Asp to Asn) and HLA-A^*^30:03N77D (with residue 77 mutated from Asn to Asp) were constructed based on wild-type HLA-A^*^30:01 and HLA-A^*^30:03, respectively, by PCR-based site-directed mutagenesis and were cloned into pET21a (+) vectors.

### Protein Expression, Refolding, and Purification

Human leukocyte antigen I (HLA I) complexes were obtained through *in vitro* co-refolding experiments, as previously described (22–24). Briefly, HLA-A^*^30:01, HLA-A^*^30:03, and human β_2_m expression vectors were transfected into *Escherichia coli* strain BL21(DE3) and overexpressed as inclusion bodies, after induction by treatment with 1 mM isopropyl β-D-thiogalactoside (IPTG) at 37°C. The inclusion bodies of MHC I H chain, β_2_m, and HLA peptides were refolded at a molar ratio of 1:1:3 in dilution refolding buffer (100 mM Tris HCl, pH 8.0; 400 mM L-arginine, 5 mM EDTA-Na, 5 mM glutathione, and 0.5 mM glutathione disulfide) at 4°C for at least 8 h. After refolding, proteins were concentrated and exchanged into the protein buffer (20 mM Tris-HCl, pH 8.0; 50 mM NaCl) and purified by gel filtration chromatography using a Superdex™ 200 Increase 10/300 GL column (GE Healthcare, Beijing, China).

### Crystallization and Data Collection

The sitting-drop vapor diffusion method was used to screen high-resolution crystals at both 4 and 18°C using Index, Crystal Screen I/II, and PEGRx I/II kits (Hampton Research, Aliso Viejo, CA, USA) (25). Briefly, we adjusted the HLA I complex concentrations to 5, 10, 15, or 20 mg/mL with the protein buffer. Then, 1 μL of protein solution was mixed with 1 μL of reservoir solution. The resulting solution was sealed and equilibrated against 100 μL of reservoir solution at 4 or 18°C. One to two weeks later, HLA-A^*^30:01/RT313 crystals were grown at a protein concentration of 15 mg/mL in 0.1 M sodium citrate tribasic dehydrate (pH 5.5) and 16% (w/v) polyethylene glycol (PEG) 8,000. HLA-A^*^30:03/RT313 crystals were grown at 20 mg/mL in 0.1 M HEPES (pH 7.5) and 25% (w/v) PEG 3,350 and HLA-A^*^30:03/NP44 crystals were grown at 15 mg/mL in 0.5 M ammonium sulfate, 0.1 M Tri-sodium citrate dihydrate (pH 5.6), and 1.0 M lithium sulfate monohydrate. Single crystals of HLA-A^*^30:03/MTB were grown at 20 mg/mL in 0.2 M potassium sodium tartrate tetrahydrate, 0.1 M Tri-sodium citrate dehydrate (pH 5.6), and 2.0 M ammonium sulfate. For cryoprotection, the crystals were transferred to reservoir solutions containing 20% glycerol and were then flash-frozen in a stream of gaseous nitrogen at 100 K. Diffraction data for the crystals were collected on Beamline 19U of the Shanghai Synchrotron Radiation Facility (Shanghai, China) and were processed using HKL2000 software (26).

### Structure Determination and Analysis

The structures of HLA^*^30:01/RT313, HLA^*^30:03/RT313, HLA^*^30:03/NP44, and HLA^*^30:03/MTB were determined by molecular replacement, using Collaborative Computational Project No. 4 (CCP4) software (27, 28) using the HLA-A^*^03:01 crystal structure (Protein Data Bank (PDB) code: 3RL1) (13) as the search model. Extensive model building was performed manually using the Crystallographic Object-Oriented Toolkit (COOT) program (29) and restrained refinement was performed using the Refinement of Molecular Structures (REFMAC5) program (30). The stereochemical quality of the final model was assessed with the program, REFINE, in Phenix (31). All figures were generated using PyMOL (http://www.pymol.org/).

### Thermostability Measurements Using Circular Dichroism

Circular dichroism (CD) was used to evaluate the thermostability of HLA I complexes, as previously reported (32). Briefly, the complexes were diluted to 200 μg/mL in protein buffer. The CD spectra at 218 nm were measured on a Chirascan spectrometer (Applied Photophysics, Leatherhead, UK) using a thermostatically controlled cuvette at temperature intervals of 0.2°C and a rate of 1°C/min between 20 and 90°C. The proportion of denatured protein was calculated from the mean residue ellipticity (u) using a standard method:

$$\begin{array}{l} \text{Fraction unfolded}\left(\text{\%}\right)=\left(\theta -\theta_{N}\right)/\left(\theta_{U}-\theta_{N}\right) \end{array}$$

where θ_*N*_ and θ_*U*_ are the mean residue ellipticity values in the fully folded and fully unfolded states, respectively. The midpoint transition temperature (*T*_m_) was calculated using data from the denaturation curves in the program Origin 8.0 (OriginLab, Northampton, MA, USA).

### Sequence Logo Plot of Amino Acid Motifs

We used one of most widely used MHC peptide binding prediction tools, NetMHCpan (http://www.cbs.dtu.dk/services/NetMHCpan/), to predict which peptides from the M (GenBank: AJD12325.1), NP (GenBank: AJJ90589.1), and PB1 (GenBank: ARG44354.1) proteins of the avian influenza virus strain H7N9, for potential presentation by HLA-A^*^30:01 and HLA-A^*^30:03. We set the score cut-off at 0.3 nM and selected 215 candidate peptides for binding to HLA-A^*^30:01 and 50 candidate peptides for binding to HLA-A^*^30:03. We then used Weblogo (http://weblogo.berkeley.edu/logo.cgi) to obtain the amino acid motifs of the F pockets of HLA-A^*^30:01 and HLA-A^*^30:03.

### Protein Structure Accession Numbers

The accession numbers of HLA-A^*^30:01/RT313, HLA-A^*^30:03/RT313, HLA-A^*^30:03/NP44, and HLA-A^*^30:03/MTB in the PDB are 6J1W, 6J1V, 6J2A, and 6J29, respectively.

## Results

### Peptide-Binding Features of HLA-A^*^30:01 and HLA-A^*^30:03

HLA I alleles in the A1 and A3 supertypes are diverse. Based on amino acid sequence alignment of typical alleles, including HLA-A^*^30:01, HLA-A^*^30:03, HLA-A^*^03:01, HLA-A^*^11:01, HLA-A^*^31:01, HLA-A^*^33:03, HLA-A^*^68:01, HLA-A^*^01:01, HLA-A^*^26:01, and HLA-A^*^26:02, we found four amino acids (Ser9, Ser17, Glu114, and His116) in HLA-A^*^30:01 and HLA-A^*^30:03 that were different from other A1 and A3 supertype alleles (Supplemental Figure 1). Within these residues, Ser9 is located in the B pocket while Glu114 and His116 are in the F pocket of the PBG and therefore, may influence peptide binding. In addition, sequence alignment of HLA-A^*^30:01 and HLA-A^*^30:03 indicates five amino acids which are different between these two alleles (i.e., R56G, Q70H, V76E, D77N, and W152R).

**Figure 1:**
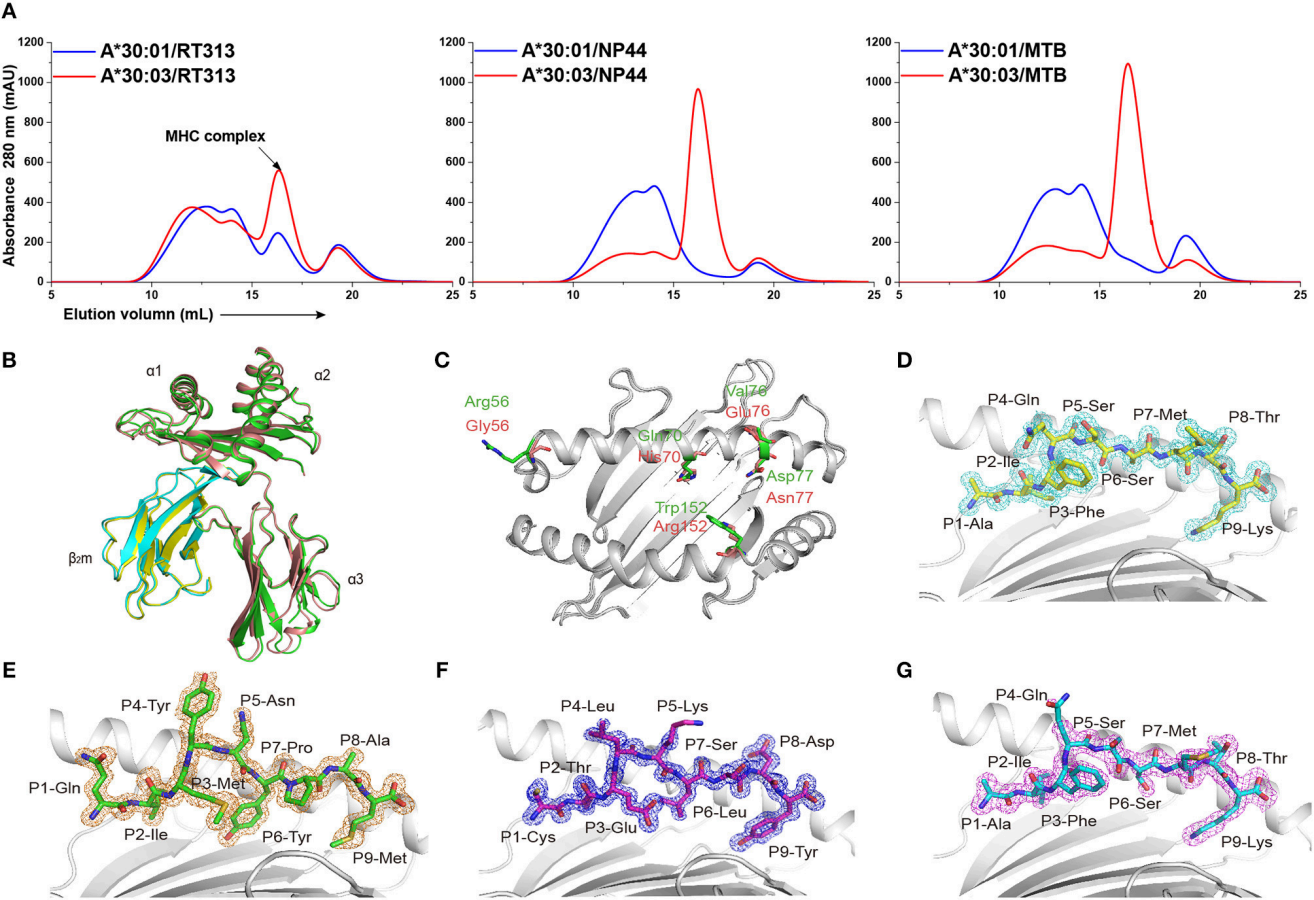
The binding capabilities and overall structures of HLA-A^*^30:01 and HLA-A^*^30:03. **(A)** Binding of RT313, NP44, and MTB to HLA-A^*^30:01 (blue) or HLA-A^*^30:03 (red) was elucidated by *in vitro* refolding assays. Peptides presented by HLA-A^*^30:01 or HLA-A^*^30:03 help their H chain and human β_2_ microglobulin to refold *in vitro*. After correct refolding, the high absorbance peaks of the HLAs with the expected molecular mass of 45 KD were eluted at an estimated volume of 16 mL on a Superdex^TM^ 200 10/300 GL column. **(B)** Structure alignment of HLA-A^*^30:01 (green) and HLA-A^*^30:03 (salmon) showed a similar overall conformation between the two alleles. **(C)** The amino acids that differed between HLA-A^*^30:01 (green) and HLA-A^*^30:03 (salmon) are shown as sticks. The authentic conformations of peptides RT313 **(D)** in the HLA-A^*^30:01 grooves and MTB **(E)**, NP44 **(F)**, and RT313 **(G)** in the HLA-A^*^30:03 grooves are shown in the 2Fo-Fc electron density maps contoured at 1.0 s. The peptide from one molecule in the asymmetric unit of each of the two structures is shown as a representative example. The residues in the peptides are labeled as individual amino acids with their position numbers.

Previous studies have shown that RT313 (AIFQSSMTK) is presented by HLA-A^*^11:01, HLA-A^*^03:01, and HLA-A^*^68:01, which all belong to the A3 supertype (13, 19), whereas NP44 (CTELKLSDY) is a typical epitope presented by the A1 supertype allele, HLA-A^*^01:01 (17). Meanwhile, a recombinant MHC class I molecule renature assay has shown that MTB (QIMYNYPAM) is presented by HLA-A^*^30:01 (18). Here, we found that RT313 also formed stable complexes with both HLA-A^*^30:01 and HLA-A^*^30:03 through co-refolding assays (Table 1, Figure 1A). In contrast, NP44 could bind HLA-A^*^30:03 but not HLA-A^*^30:01 *in vitro* (Table 1, Figure 1A). Similarly, no binding was observed between MTB and HLA-A^*^30:01 in co-refolding experiments. In contrast, MTB could be presented by HLA-A^*^30:03 (Table 1, Figure 1A). These results may indicate that HLA-A^*^30:03 can not only bind peptides cross-presented by A1 supertype alleles, but can also accommodate peptides cross-presented by A3 supertype alleles, with A1A3 supertype features. However, HLA-A^*^30:01 could only bind peptides cross-presented by A3 supertype alleles. Although peptide RT313 was able to bind both HLA-A^*^30:01 and HLA-A^*^30:03, circular dichroism (CD) assays showed that HLA-A^*^30:01/RT313 (with a mean *T*_*m*_ of 45.4°C) was much more stable than HLA-A^*^30:03/RT313 (with a mean *T*_*m*_ of 37.3°C) (Supplemental Figure 2).

To further confirm the peptide-binding motifs of HLA-A^*^30:01 and HLA-A^*^30:03, we mutated the PΩ-Met residue of MTB to different A1 supertype peptide motif-preferred residues (Tyr, Phe, Ile, Leu, and Val), A3 supertype binding motif residues (Arg and Lys), and small hydrophilic amino acids (Ser and Thr) (Table 2). We found that HLA-A^*^30:03 could present MTB-M9Y, MTB-M9F, MTB-M9I, MTB-M9V, and MTB-M9L peptides with A1 supertype binding motifs and could also present the MTB-M9K peptide with A3 supertype binding motifs. However, it could not present MTB-M9R (Table 2, Supplemental Figure 3). Peptides with small hydrophilic amino acid mutations could also be presented by HLA-A^*^30:03 (Table 2, Supplemental Figure 3). However, HLA-A^*^30:01 could present only those mutated peptides that contained A3 supertype binding motifs MTB-M9K and MTB-M9R (Table 2, Supplemental Figure 3).

There are 950 peptides available in the Immune Epitope Database (IEDB) (http://www.iedb.org) which are reported to bind HLA-A^*^30:01. However, only 23 peptides were published with defined assays, For 15 potential HLA-A^*^30:01-binding peptides with aliphatic or aromatic amino acids as PΩ residues (Table 3). we verified the binding of the peptides with HLA-A^*^30:01 using the co-refolding assay. HLA-A^*^30:01 did not show any binding capacity to any of these 15 peptides. In contrast, five peptides (IEDB1, IEDB2, IEDB9, IEDB11, and IEDB15) with Leu, Ser, Ile, Val, and Tyr at PΩ residue could help HLA-A^*^30:03 renature *in vitro* (Supplemental Figures 4A–E). These results also support that HLA-A^*^30:01 possesses more features of A3 supertype compared to HLA-A^*^30:03.

### The Overall Structure of HLA-A^*^30:01 and HLA-A^*^30:03

To explore the structural basis of the different peptide-binding characteristics of HLA-A^*^30:01 and HLA-A^*^30:03, we determined the structures of HLA-A^*^30:01/RT313, HLA-A^*^30:03/RT313, HLA-A^*^30:03/NP44, and HLA-A^*^30:03/MTB at 1.5, 2, 1.4, and 1.6 Å, respectively (Table 4). The overall structures of these four complexes showed typical MHC I conformations (Figure 1B). The root-mean-square deviation (RMSD) of the overall structures of HLA-A^*^30:01 and HLA-A^*^30:03 was 0.591, which indicated that these two molecules were extremely similar. Five amino acids differed between HLA-A^*^30:01 and HLA-A^*^30:03 and these were mainly located at the α1α2 domains of the heavy chains and residues 70, 77, and 152 in the PBG, which may influence peptide binding (Figure 1C). The electron densities for these three peptides were well-defined into the PBG of HLA-A^*^30:01 and HLA-A^*^30:03, which indicated that the conformations of these four structures were stable (Figures 1D–G).

**Table 4:** X-ray diffraction data processing and refinement statistics.

**Statistics**	A*30:01/RT313	A*30:03/RT313	A*30:03/NP44	A*30:03/MTB
**Data processing**				
Space group	P2_1_2_1_2_1_	P2_1_2_1_2_1_	C2	C2
**Cell parameters** **(Å)**				
a (Å)	79.27	52.26	155.74	155.82
b (Å)	72.03	72.13	79.42	79.49
c (Å)	78.01	124.68	44.74	44.82
α (°)	90.00	90.00	90.00	90.00
β (°)	90.00	90.00	93.74	94.04
γ (°)	90.00	90.00	90.00	90.00
Resolution range (Å)	50.0–1.5 (1.55–1.50)^a^	50.0–2.0 (2.07–2.00)	50.0–1.4 (1.45–1.40)	50.0–1.6 (1.66–1.60)
Total reflections	905897	237620	723559	493978
Unique reflections	71391	32751	106161	72173
Completeness (%)	99.1 (91.8)	99.8 (99.9)	99.3 (99.9)	99.7 (100.0)
R_merge_ (%)^b^	6.2 (45.9)	10.3 (58.0)	6.9 (64.3)	6.3 (39.2)
I/σ	36.7 (4.4)	19.3 (3.5)	28.3 (3.0)	26.7 (5.4)
**Refinement**				
R_work_ (%)^c^	18.82	20.23	18.17	17.94
R_free_ (%)	20.55	21.71	19.54	19.66
** RMSD**				
Bond lengths (Å)	0.013	0.008	0.008	0.013
Bond angles (°)	1.46	1.11	1.20	1.48
Average B factor (Å^2^)	18.95	27.98	19.07	17.33
** Ramachandran plot quality**				
Most favored (%)	98.94	98.40	98.94	99.20
Allowed (%)	1.06	1.60	1.06	0.80
Disallowed (%)	0	0	0	0

a *Values in parentheses are those for the highest resolution shell*.

b *R_merge_ = ∑hkl∑i|I_i_−〈I〉|∑hkl∑i I_i_, where I_i_ is the observed intensity, and 〈I〉is the average intensity of multiple observations of symmetry-related reflections*.

c *R = ∑hkl||F_obs_|−k|F_cal_||/∑hkl|F_obs_|, where R_free_ is calculated for a randomly chosen 5% of reflections, and R_work_ is calculated for the remaining 95% of reflections used for structure refinement*.

### Similar PΩ-Lys Anchoring of HLA-A^*^30:01- and HLA-A^*^30:03-Binding Peptides

**Figure 2:**
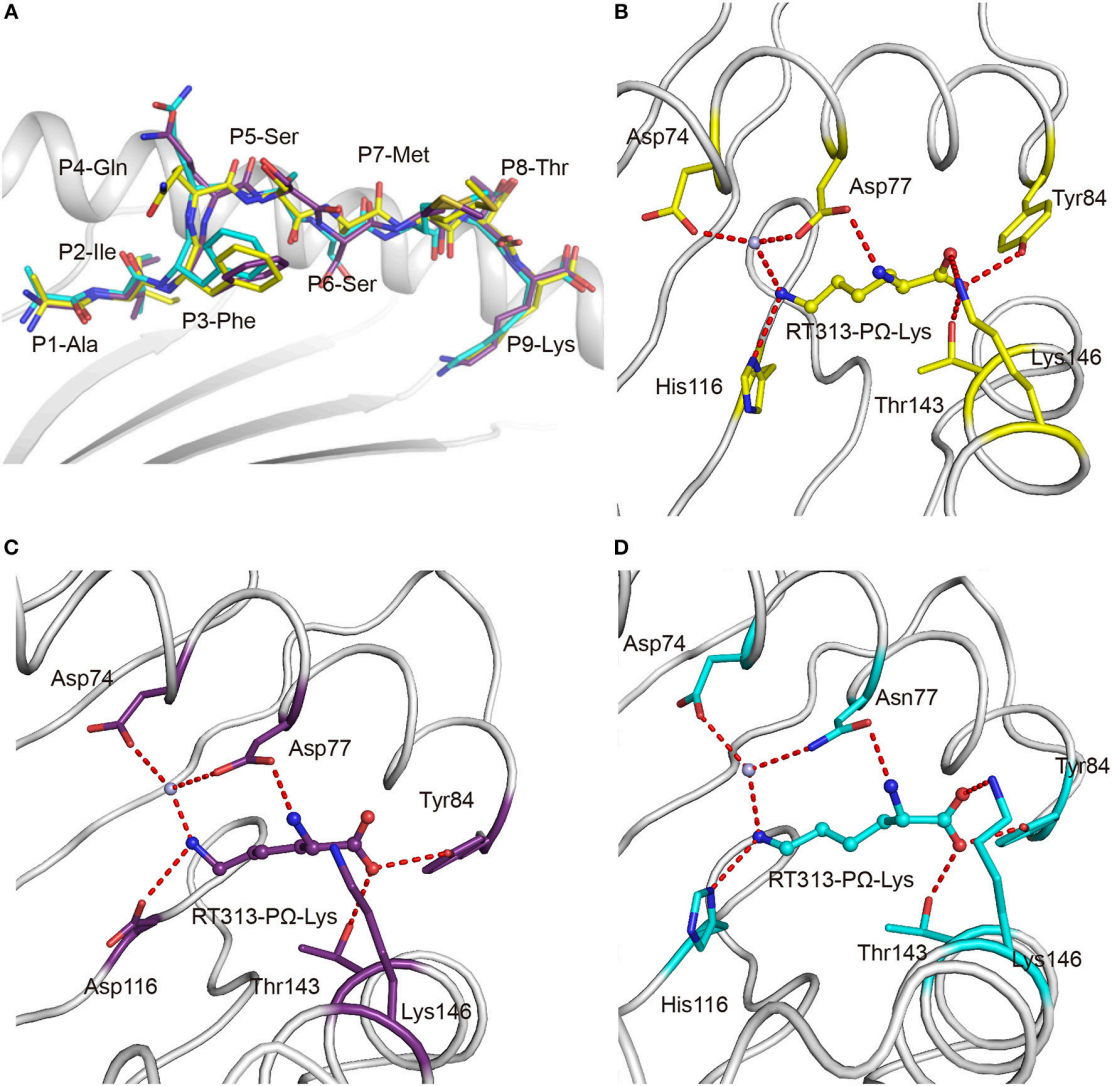
Similar anchoring modes of A3 supertype peptide in the F pockets of HLA-A^*^30:01 and HLA-A^*^30:03. **(A)** Superposition of RT313 bound to HLA-A^*^30:01 (yellow), HLA-A^*^30:03 (cyan), or HLA-A^*^11:01 (purple). The alignment of these three structures showed a similar overall conformation for RT313 in the peptide-binding grooves. **(B–D)** The binding modes of the PΩ-Lys residue of RT313 in the F pockets of HLA-A^*^30:01 **(B)**, HLA-A^*^11:01 **(C)**, and HLA-A^*^30:03 **(D)** are shown. The major residues of the F pockets are shown as sticks while the hydrogen bonds of the F pockets are represented as red dashed lines. PΩ-Lys residues have hydrogen bonds with Asp74 through a water molecule in all three structures. Although HLA-A^*^30:01 and HLA-A^*^30:03 contain a His at position 116, which differed from HLA-A^*^11:01, which had Asp at this position, both His116 and Asp116 could form a hydrogen bond with the positively charged anchor, Lys.

The availability of the crystal structures of HLA-A^*^11:01, HLA-A^*^30:01, and HLA-A^*^30:03 with the similar A3-supertype-signature peptide, RT313, provided the opportunity to compare their cross-presentation features. Firstly, the alignment of these three structures showed that there was a similar overall conformation of the presented peptide, RT313, in the PBGs (Figure 2A). Our crystallographic data showed that although the key residues at position 74, 77, 114, and 116 are different in the F pockets of HLA-A^*^11:01, HLA-A^*^30:01, and HLA-A^*^30:03, the binding modes of the PΩ-Lys residue of RT313 in the F pockets of these three molecules were quite similar. The NZ-atom of the PΩ-Lys residue had hydrogen bonds with Asp74, through a water molecule in HLA-A^*^11:01, HLA-A^*^30:01, and HLA-A^*^30:03 (Figures 2B–D). Meanwhile, HLA-A^*^30:01 and HLA-A^*^30:03 possess a His at position 116, differed from HLA-A^*^11:01, which has an Asp at this position. Similar to the direct hydrogen bond between Asp116 in HLA-A^*^11:01 and PΩ-Lys in RT313 (Figure 2C), His116 in HLA-A^*^30:01 and HLA-A^*^30:03 could also form hydrogen bonds with the positively-charged anchor Lys (Figures 2B–D). Although residue 77 of HLA-A^*^30:01 (Asp77) and HLA-A^*^30:03 (Asn77) were different, the NZ-atom of PΩ-Lys also formed direct hydrogen bonds with Asp77 in HLA-A^*^11:01 and HLA-A^*^30:01 and with Asn77 in HLA-A^*^30:03 (Figures 2B–D).

### Special A1A3 Supertype Features in the F Pocket of HLA-A^*^30:03

**Figure 3:**
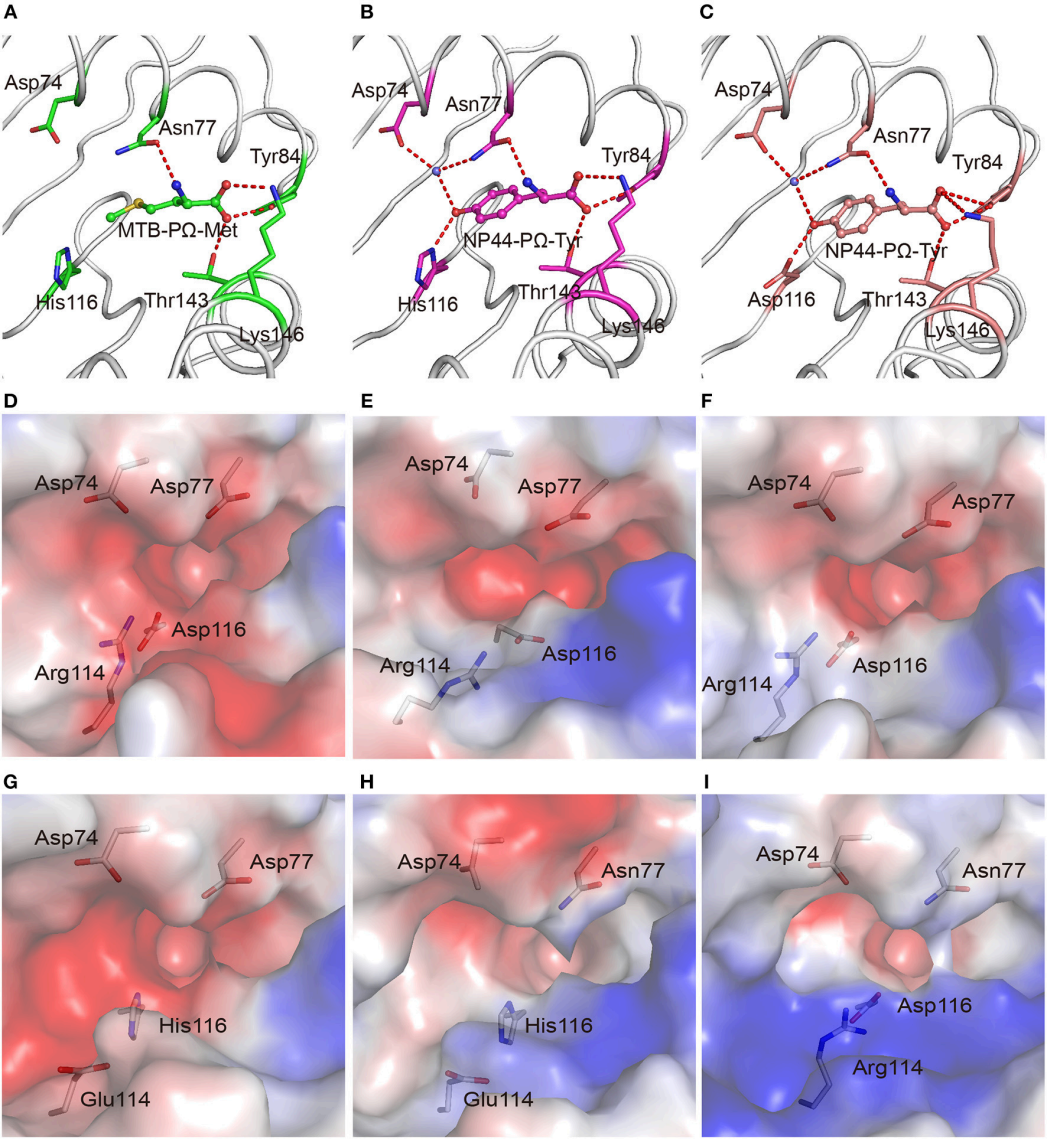
The F pocket of HLA-A^*^30:03 with A1A3 supertype features. The binding modes of the PΩ-Met residue of MTB in the F pocket of HLA-A^*^30:03 **(A)**, and the PΩ-Tyr residue of NP44 in the F pocket of HLA-A^*^30:03 **(B)**, and HLA-A^*^01:01 **(C)**. The major residues of the F pocket are shown as sticks. The hydrogen bonds of the F pocket are represented as red dashed lines. PΩ-Lys residues had hydrogen bonds with Asp74 through a water molecule in all three structures. Both His116 in HLA-A^*^30:03 and Asp116 in HLA-A^*^01:01 could form hydrogen bonds with the PΩ residues of MTB and NP44. Vacuum electrostatic surface potential of the F pockets of HLA-A^*^03:01 **(D)**, HLA-A^*^68:01 **(E)**, HLA-A^*^11:01 **(F)**, HLA-A^*^30:01 **(G)**, HLA-A^*^30:03 **(H)**, and HLA-A^*^01:01 **(I)**. Residues 74, 77, 114, and 116 are shown as sticks under the vacuum electrostatic surface. The F pocket of HLA-A^*^30:01 had a strong negative charge, which was extremely similar to the F pockets of the A3 supertype alleles, HLA-A^*^03:01, HLA-A^*^68:01, and HLA-A^*^11:01. Arg114 of HLA-A^*^01:01 pointed to the “mouth” of the F pocket to form a strong positively charged environment at the “mouth” of the F pocket. The side chain of Glu114 in HLA-A^*^30:03 was shorter than that of Arg114. The strength of the negative charge of the F pocket of HLA-A^*^30:03 was between those in A3 and A1 supertype alleles.

In addition to binding peptides through PΩ-Lys, as do other A3 supertype members such as HLA-A^*^30:01 and HLA-A^*^11:01, HLA-A^*^30:03 could also bind peptides with hydrophobic residues as PΩ anchors. The PΩ residues of MTB (Met) and NP44 (Tyr) could also form stable hydrogen bonds with the residues in the F pocket of HLA-A^*^30:03 (Figures 3A,B). Furthermore, in the structure of HLA-A^*^30:03/NP44, additional water molecules can help the PΩ-Tyr of peptide NP44 bind to Asp74 and Asn77 in an α helix, to enable stable peptide binding (Figure 3B), quite similar as in the structure of HLA-A^*^01:01/ NP44 (Figure 3C).

We compared the charge of the F pocket of HLA-A^*^30:01 and HLA-A^*^30:03 with the charge of the F pocket of the typical A3 supertype molecules, HLA-A^*^03:01, HLA-A^*^68:01, and HLA-A^*^11:01, and the typical A1 supertype molecule HLA-A^*^01:01 (Figures 3D–I). In HLA-A^*^03:01, HLA-A^*^68:01, and HLA-A^*^11:01, residues 74, 77, and 116 were the negatively charged acidic amino acid, Asp, which resulted in F pockets with a strong negative charge (Figures 3D–F). In HLA-A^*^30:01, even though residue 116 was His, not Asp, the F pocket still had strong negative charge (Figure 3G), thereby allowing HLA-A^*^30:01 to bind positively charged amino acids, such as Lys and Arg. This also suggested that, when His/Asp116 is the only difference between HLA-A^*^30:01 and HLA-A^*^30:03, the residue in this position has little impact on the charge of the F pocket. In contrast, residue 77 was different between HLA-A^*^01:01 (Asn77) and the A3 supertype alleles (Asp77). Asn has a weaker negative charge than Asp and therefore, the HLA-A^*^01:01 F pocket has a weaker negative charge than the A3 supertype F pockets (Figure 3I). The difference in the charge of the F pockets between the A1 and A3 supertypes was one of the main reasons for their different binding motifs. This suggests that residue 77 plays a key role in the definition of different supertypes.

Although residue 77 in HLA-A^*^30:03 was also Asn, its F pocket was able to bind amino acids belonging to both A1 and A3 supertypes. Comparing the charge of the F pocket between HLA-A^*^30:03 and HLA-A^*^01:01, we found that the “mouth” of the HLA-A^*^01:01 F pocket had a stronger positive charge than that of the HLA-A^*^30:03 F pocket (Figures 3H,I). Upon further analysis, we found that Arg114 of HLA-A^*^01:01 pointed to the “mouth” of the F pocket to form a strong positively charged environment thereby preventing positively charged amino acids, such as Lys and Arg, from inserting into the F pocket (Figure 3I, Supplemental Figure 5A). However, in HLA-A^*^30:03, Glu114 did not form the F pocket because its side chain is much smaller than that of Arg (Supplemental Figure 5B). The strength of the negative charge of the HLA-A^*^30:03 F pocket was between that of the A3 supertype and A1 supertype alleles (Figure 3H). This allowed the F pocket of HLA-A^*^30:03 to accommodate both the basic amino acids preferred by the A3 supertype and the aromatic and large hydrophobic amino acids preferred by the A1 supertype.

### Polymorphic Residues Distinguish the Peptide Presentation of HLA-A^*^30:03 From HLA-A^*^01:01 in the A1 Supertype

**Figure 4:**
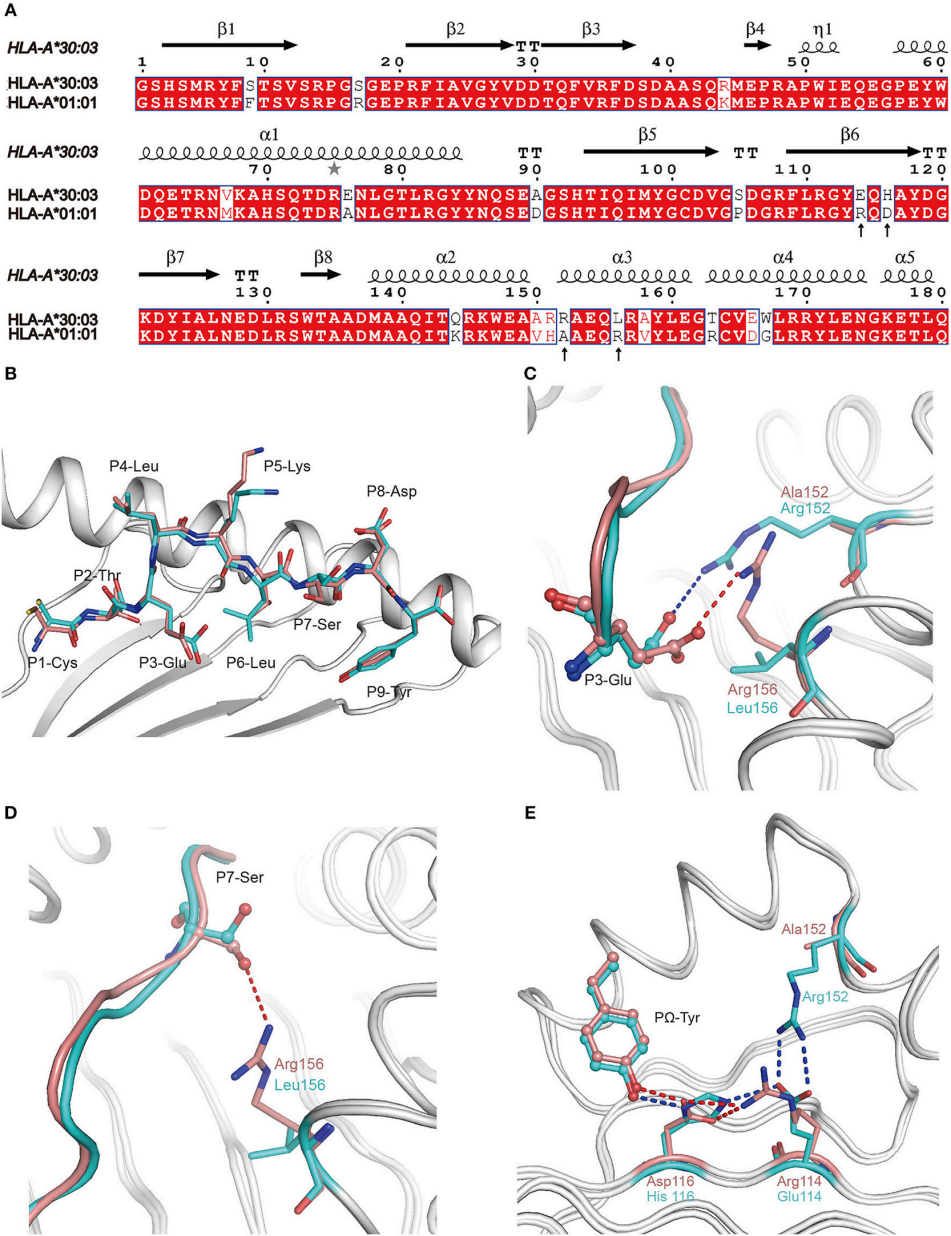
The contribution of polymorphic amino acids to the different construction of NP44 presented by HLA-A^*^30:03 and HLA-A^*^01:01 and to the recognition of TCRs. **(A)** Structure-based sequence alignment of HLA-A^*^30:03 and HLA-A^*^01:01, addressing the α1 and α2 domains. Cylinders indicate α-helices and black arrows indicate β-strands. Amino acids highlighted in red are completely conserved and those in blue boxes are highly (>80%) conserved. Sequence alignment was generated with Clustal X (33) and ESPript (34). **(B)** Superposition of NP44 presented by HLA-A^*^30:03 and HLA-A^*^01:01. The overall conformations of NP44 in HLA-A^*^30:03 and HLA-A^*^01:01 were very similar. However, P3-Glu, P5-Lys, and P7-Ser showed slightly different conformations in these two structures. **(C)** The different conformations of P3-Glu of NP44 presented by HLA-A^*^30:03 (cyan) and HLA-A^*^01:01 (lemon). The hydrogen bonds between P3-Glu and residues in HLA-A^*^30:03 are represented as blue dashed lines and those with residues in HLA-A^*^01:01 are represented as red dashed lines. **(D)** The different conformations of P7-Ser of NP44 presented by HLA-A^*^30:03 (cyan) and HLA-A^*^01:01 (lemon). The hydrogen bonds between P7-Ser and Arg156 in HLA-A^*^01:01 are represented as red dashed lines While, Leu156 in HLA-A^*^30:03 could not form hydrogen bonds with P7-Ser. **(E)** Different hydrogen bond networks of PΩ-Tyr of NP44 with residues 114 and 116 of HLA-A^*^30:03 and HLA-A^*^01:01. Hydrogen bonds are represented as blue dashed lines in HLA-A^*^30:03/NP44 and as red dashed lines in HLA-A^*^01:01/NP44.

All of the 18 amino acid polymorphisms between HLA-A^*^30:03 and HLA-A^*^01:01 were located at the α1 and α2 domains (Figure 4A). Furthermore, most of these polymorphic residues were located at the PBG, which may contribute to their subtly different peptide presentations and the HLA-restriction of TCR-recognition. Among the polymorphic residues between HLA-A^*^30:03 and HLA-A^*^01:01, amino acids at positions 76, 150, 151, 152, and 163 were located in the TCR-contacting regions. Polymorphic residues included negatively charged Glu vs. small Ala at position 76, a small Val vs. smaller Ala at position 150, longer Arg vs. shorter His at position 151, positively charged Arg vs. small Ala at position 152, and nucleophilic Thr vs. positively charged Arg at position 163 of HLA-A^*^30:03 and HLA-A^*^01:01, respectively (Figure 4A) (35–37).

Alignment analysis of NP44 presented by HLA-A^*^01:01 and HLA-A^*^30:03 showed a similar overall conformation of the presented peptides in the PBGs of the two alleles (Figure 4B). However, the side chain of P3-Glu, P5-Lys, and P7-Ser point to different orientations in HLA-A^*^01:01/NP44 and HLA-A^*^30:03/NP44, respectively (Figure 4B). Further analysis revealed that Arg152 of HLA-A^*^30:03 formed a salt bridge with P3-Glu. However, in HLA-A^*^01:01, Arg156 also formed a salt bridge with P3-Glu, which resulted in a subtle difference in the conformation of P3-Glu (Figure 4C). In addition, Arg156 of HLA-A^*^01:01 could also form a hydrogen bond with P7-Ser. However, no hydrogen bond was seen between Leu156 and P7-Ser in HLA-A^*^30:03 (Figure 4D). Even though the PΩ-Tyr side chain of NP44 could form a hydrogen bond with both the positively charged His116 in HLA-A^*^30:03 and the negatively charged Asp116 in HLA-A^*^01:01, the hydrogen bond networks of residue 114, 116, and the PΩ-Tyr of NP44 were different (Figure 4E). In HLA-A^*^30:03, the negatively charged Glu114 could form a salt bridge with the positively charged His116 and Arg152. For HLA-A^*^01:01/NP44, residue 114 was a positively charge Arg, which formed a salt bridge with Asp116 to stabilize its conformation. Ala152 is a small amino acid which was unable to form a direct contact with Arg114.

### The Key Contribution of Residue 77 to the Peptide-Binding Motifs of HLA Supertypes

**Figure 5:**
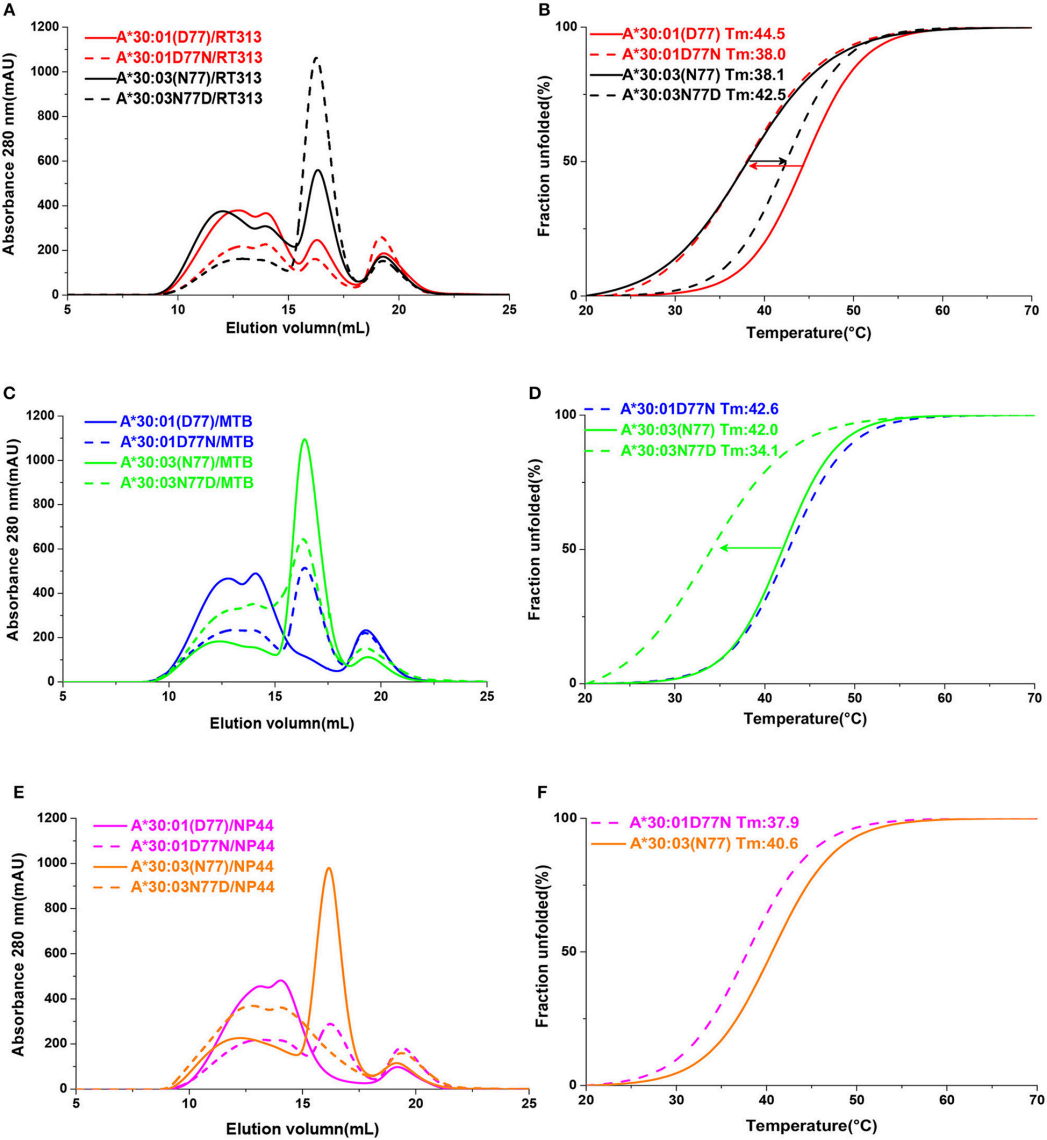
The binding capabilities of peptides with HLA-A^*^30:01, HLA-A^*^30:03, and the mutants HLA-A^*^30:01D77N, or HLA-A^*^30:03N77D. Gel filtration chromatograms **(A)** and Thermal stabilities tested by circular dichroism spectroscopy **(B)** of HLA-A^*^30:01/RT313 (red solid line), HLA-A^*^30:03/RT313 (black solid line), HLA-A^*^30:01D77N/RT313 (red dashed line), and HLA-A^*^30:03N77D/RT313 (black dashed line). **(C,D)** Gel filtration chromatograms **(C)** and Thermal stabilities **(D)** of HLA-A^*^30:01/MTB (blue solid line), HLA-A^*^30:03/MTB (green solid line), HLA-A^*^30:01D77N/MTB (blue dashed line), and HLA-A^*^30:03N77D/MTB (green dashed line). **(E,F)** Gel filtration chromatograms **(E)** and Thermal stabilities **(F)** of HLA-A^*^30:01/NP44 (magenta solid line), HLA-A^*^30:03/NP44 (orange solid line), HLA-A^*^30:01D77N/NP44 (magenta dashed line), and HLA-A^*^30:03N77D/NP44 (orange dashed line). The arrow shows the direction of the change in thermal stability after mutagenesis.

In order to confirm that residue 77 was the key position to determine the different binding motifs between HLA-A^*^30:01 and HLA-A^*^30:03, we mutated Asp77 to Asn77 in HLA-A^*^30:01 (mutant, mutated HLA-A^*^30:01D77N) and Asn77 to Asp 77 in HLA-A^*^30:03 (mutant, mutated HLA-A^*^30:03N77D). As in the binding assays, peptide RT313 could be presented by both mutated HLA molecules (Figure 5A). However, in comparison to wild type HLA-A^*^30:01, the binding capacity of HLA-A^*^30:01D77N to RT313 decreased. In contrast, the binding capacity of HLA-A^*^30:03N77D to RT313 increased in comparison to wild type HLA-A^*^30:03 (Figure 5B). Peptide MTB, which has an A1 supertype-binding peptide-featured PΩ anchor, could not bind HLA-A^*^30:01, but after the introduction of the D77N mutation, HLA-A^*^30:01D77N was able to bind MTB (Figures 5C,D). We found the melting temperature (*T*_m_) of HLA-A^*^30:01D77N/MTB to be 42.6°C. In contrast, even though MTB could still be presented by HLA-A^*^30:03N77D, the binding capacity decreased significantly after mutagenesis (Figure 5C). Similarly, peptide NP44 could be presented by wild type HLA-A^*^30:03, but not by HLA-A^*^30:01 (Figures 5E,F). Mutant HLA-A^*^30:03N77D lost the ability to bind with NP44, whereas HLA-A^*^30:01D77N bound NP44 with high capacity (Figures 5E,F). The peptide-binding ability of the F pocket of HLA-A^*^30:01 and HLA-A^*^30:03 shifted in opposite directions after mutation of residue 77. These results indicate that residue 77 is the key position involved in determining the different binding motifs between HLA-A^*^30:01 and HLA-A^*^30:03.

**Figure 6:**
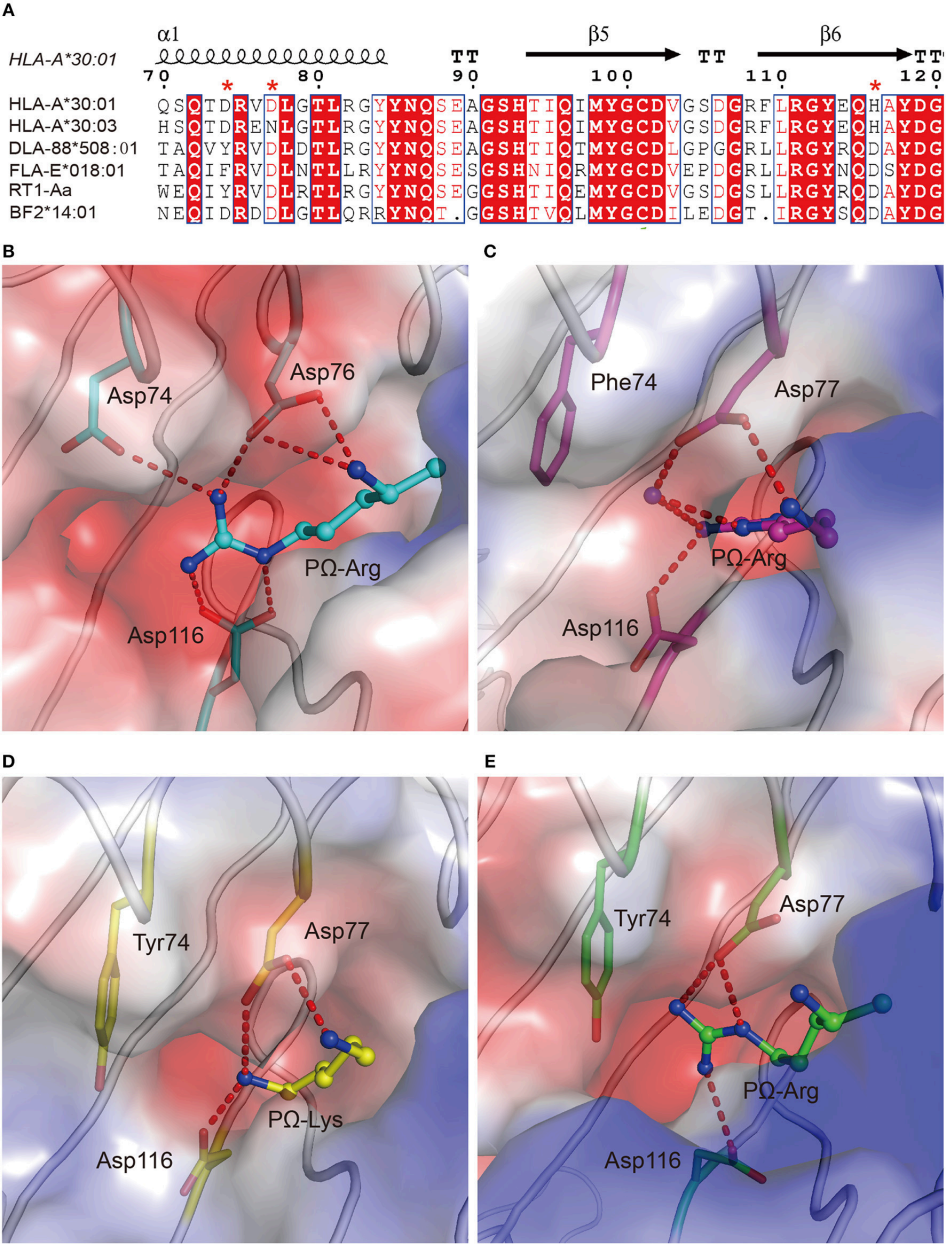
The preference of peptides with PΩ Lys/Arg by MHC I with Asp77 in non-human animals. **(A)** Structure-based sequence alignment of HLA-A^*^30:01, HLA-A^*^30:03, DLA-88*508:01, FLA-E*018:01, RT1-A^a^, and BF2*14:01. Cylinders indicate α-helices and black arrows indicate β-strands. Amino acids highlighted in red are completely conserved and those in blue boxes are highly (>80%) conserved. Residue 74, 77, and 116 were labeled with an asterisk. Vacuum electrostatic surface potential of the F pockets of chicken MHC I BF2*14:01 (PDB code: 4CW1) **(B)**, feline MHC I FLA-E*018:01 (5XMF) **(C)**, dog MHC I DLA-88*508:01 (5F1N) **(D)**, and rat MHC I RT1-A^a^ (1ED3) **(E)**. Residues 74, 77, and 116 are shown as sticks under the vacuum electrostatic surface. The hydrogen bonds between PΩ of peptides and residues 74, 77, or 116 are represented as red dashed lines.

To verify the role of Asp77 in the peptide presentation of MHC I from different vertebrates other than humans, the 1,271 MHC I crystal structures available in the PDB were retrieved and chicken MHC I BF2^*^14:01 (PDB code: 4CW1), feline MHC I FLA-E^*^018:01 (PDB code: 5XMF), dog MHC I DLA-88^*^508:01 (PDB code: 5F1N), and rat MHC I RT1-A^a^ (PDB code: 1ED3) prefer peptides with positive charged Lys or Arg residue at the PΩ residue (38–41). Among these MHC Is, residue 74 and residue 116 were diversified. In contrast, residues at position 77 of these four MHC class I molecules retain a conserved Asp (Figure 6A). The F pockets of BF2^*^14:01, FLA-E^*^018:01, DLA-88^*^508:01, and RT1-A^a^ are negatively charged (Figures 6B–E). It indicated that Asp77 in the F pocket of MHC I from different vertebrates may play a key role in the binding of peptides with Lys or Arg at PΩ.

### The Peptide Presentation of HLA-A^*^30 Alleles Represented by HLA-A^*^30:01 and HLA-A^*^30:03

In addition to HLA-A^*^30:01 and HLA-A^*^30:03, HLA-A^*^30 serotype also includes alleles e.g., HLA-A^*^30:02 and -A^*^30:04 covering a board population worldwide. There are totally 134 HLA-A^*^30 serotype alleles available in the International Immunogenetics Information System (IMGT, https://www.ebi.ac.uk/ipd/imgt/hla). Among these HLA alleles, residues 74, 114, and 116 of A^*^30 serotype are highly conserved, while residue 77 is either Asp or Asn (Supplemental Tables 1, 2). A^*^30 serotype-carrying population are mainly distributed in Asia, Africa, and North America (http://www.allelefrequencies.net/default.asp). The frequencies of HLA-A^*^30:01 among the specific ethnic groups in these areas ranges from 3 to 16% (Supplemental Figure 6A). HLA-A^*^30:03-carrying populations are mainly located in Yaoundé, Cameroon, with a low frequency of 1.1% (Supplemental Figure 6B). However, HLA-A^*^30:02 and -A^*^30:04 can be classified into the similar group as HLA-A^*^30:03 and loaded the similar key residues in PBGs for the A1A3 supertype. HLA-A^*^30:02 and HLA-A^*^30:04-carrying population are mainly located in Africa. The highest frequency (23.3%) of HLA-A^*^30:02 occurs in Lusaka, Zambia. HLA-A^*^30:04 occurs in 27.2% of the population of the Mbenzele Pygmies from the Central African Republic (Supplemental Figure 6B). Therefore, even though the coverage of HLA-A^*^30:03 is not high in the general worldwide population, the related alleles HLA-A^*^30:02 and HLA-A^*^30:04 are popular, not only in Africa but also in other continents.

## Discussion

Supertype definitions provide a pivotal tool for peptide screening in vaccine development and immune intervention strategies. Currently, the classification of some HLA I alleles into defined supertypes is in debate, but structural determination will provide visualized evidence that will aid our understanding of supertype definitions. In this study, we found that HLA-A^*^30:01 possesses more features of A3 supertype compared to HLA-A^*^30:03, and the peptide-binding motifs of HLA-A^*^30:03 belonged to the A1A3 supertype. These conclusions were made based on the crystal structure determination of four peptide/HLA complexes and the results of a series of biochemical investigations *in vitro*.

**Figure 7:**
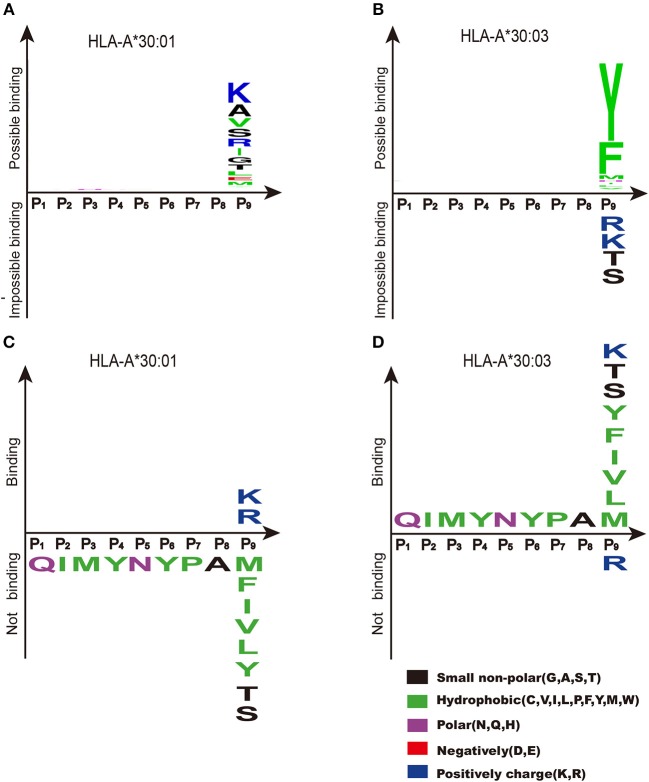
Binding motifs of F pockets of HLA-A^*^30:01 and HLA-A^*^30:03. Binding motifs of the F pocket of HLA-A^*^30:01 **(A)** and HLA-A^*^30:03 **(B)** as predicted by NetMHCpan (http://www.cbs.dtu.dk/services/NetMHCpan/). According to the prediction results, the F pocket of HLA-A^*^30:01 prefers residues from the A1 supertype-binding motifs (Ile, Leu, and Val), residues from A3 supertype-binding motifs (Arg and Lys), and small hydrophilic amino acids (Ala, Ser, and Thr). The F pocket of HLA-A^*^30:03 prefers residues from A1 supertype-binding motifs (Tyr and Phe). The real binding motifs of the F pockets of HLA-A^*^30:01 **(C)** and HLA-A^*^30:03 **(D)** were verified experimentally. HLA-A^*^30:01 bound to peptides with A3 supertype-binding motifs (Arg and Lys) at the PΩ position and HLA-A^*^30:03 bound to peptides with A1 supertype-binding motif residues (Tyr, Met, Phe, Ile, Leu, and Val), the A3 supertype-binding motif residue Lys, and small hydrophilic amino acids (Ser and Thr) at the PΩ position.

NetMHCpan (http://www.cbs.dtu.dk/services/NetMHCpan/) is widely used for peptide prediction (38, 42–46). Based on previous knowledge, peptide prediction using NetMHCpan showed that the PΩ motif of HLA-A^*^30:01-binding peptides was biased toward both A1 supertype-preferred residues (Ile and Leu), and A3 supertype-preferred residues (Arg and Lys) (Figure 7A). Meanwhile, the binding motif of the F pocket of HLA-A^*^30:03 preferred only those residues with A1 supertype binding motifs (Tyr and Phe) (Figure 7B). However, our experimental results indicated that HLA-A^*^30:01 bound to peptides with A3 supertype binding motifs (Arg and Lys) at the PΩ position and that HLA-A^*^30:03 bound to peptides with A1 supertype binding motifs (Tyr, Met, Phe, Ile, and Leu), A3 supertype binding motifs (Lys), and small hydrophilic amino acids (Ser and Thr) (Figures 7C,D).

Our structural studies showed that residue 77 in the F pocket may be the key residue for determining the different binding motifs of A1, A1A3, and A3 supertype alleles. When we mutated the amino acid at residue 77 of HLA-A^*^30:01 and HLA-A^*^30:03, we found that the binding capacity changed to the opposite direction for the two HLAs. One mutation, from Asp to Asn at position 77 in HLA-A^*^30:01, enabled it to bind to the A1 supertype-binding peptides MTB and NP44. This indicated that Asp at position 77 restricted the binding of the F pocket of HLA-A^*^30:01 to the positively charged residues of the binding peptides. When Asp77 was mutated to Asn, the anchor residue could be expended to other hydrophobic residues (e.g., Met or Tyr) in MTB and NP44. However, even though the binding capacity decreased when Asn77 of HLA-A^*^30:03 was mutated to Asp77, the mutant HLA could still present MTB. This indicated that, although residue 77 was the main factor influencing the binding motif of the F pocket, it was not the only reason for the restricted peptide presentation.

The cross-presentation of peptides from both A1 and A3 supertypes means that HLA-A^*^30:03 belongs to a special supertype, A1A3. Interestingly, the peptide-binding modes of HLA-A^*^30:03 possess unique features that are different from typical A1 and A3 supertype alleles. In contrast to HLA-A^*^01:01, different residues at position 114 resulted in different binding motifs of HLA-A^*^30:03 by influencing the charge surrounding of the F pocket. Although residue 116 can form a stable hydrogen bond with the PΩ residue of the peptide, the binding motifs of the F pocket did not change when Asp116 was mutated to His116. In HLA-A^*^01:01, Arg114 pointed to the “mouth” of the F pocket to form a strong positively charged environment, which may repel positive amino acids, such as Lys and Arg. However, in HLA-A^*^30:03, Glu114 did not participate in the formation of the F pocket. The strength of the negative charge of the HLA-A^*^30:03 F pocket was between that of the A3 and A1 supertype alleles. Therefore, HLA-A^*^30:03 has A1A3 supertype peptide binding characteristics. In contrast to the typical A3 supertype alleles, HLA-A^*^30:03 could not present MTB when the PΩ residue was mutated to an Arg. One possible reason for this is that Arg has a stronger positive charge than Lys, while Asn77 and Glu114 gives a weaker negative charge in the F pocket of HLA-A^*^30:03 than that of A3 supertype alleles. The positive charge of Arg was too strong for it to insert into the F pocket of HLA-A^*^30:03. Based on these results, we can also predict the supertype of other similar HLA alleles (Supplemental Table 1).

Screening of immunogenic peptides which are presented by specific MHC I molecules is crucial for the development of vaccines for infection diseases or cancer immunotherapy and for T-cell immune response evaluation (45–51). Recently, several pre-clinical and clinical studies have shown that vaccines targeting predicted personal tumor neoantigens are feasibility, safety, and immunogenicity (45, 50, 52–55). Adoptive T cell therapy that specifically recognize neoantigens has mediated substantial objective clinical regressions in patients with melanoma and metastatic breast cancer (56–58). In our previous work, even one immunogenic CD8^+^ T-cell peptide could stimulate a strong T-cell immune response *in vivo* and could reduce virus shedding during challenge in mice (25). In chickens, immunogenic CD8^+^ T-cell peptides have been identified from the Rous sarcoma virus and infectious bursal disease virus and these peptides had immune-protective functions (59, 60). Due to an unusually large PBG in the MHC class I molecule, BF2^*^21:01, compared with BF2^*^04:01, BF2^*^21:01 is able to present a greater diversity of peptides than BF2^*^04:01. Therefore, chickens with the BF2^*^21:01 allele possess stronger antiviral abilities than those with the BF2^*^04:01 allele (59, 60). However, to rapidly and precisely identify mutated epitope targets in individual patients still remains a daunting technical challenge. This indicates that binding motif-based HLA supertype classification could offers a simply and precisely way to characterize the peptides presented by a certain individual HLA allele and to understanding the disease susceptibility from the view of immunity. However, HLA alleles with single amino acid difference outside the pockets may not influence the binding motifs, but can also impact the disease status of patients by disturbing the TCR recognition (25, 61).

Cross-reactive CD8^+^ T cells broadly exist among the population and play a pivotal role in the defense against viral infections. A number of cross-presented T-cell epitopes have been identified thus far (32, 62–64). Most of these are cross-recognized by the same HLA supertype because they have similar binding motifs. We previously reported that the HIV peptide, RT313, can be cross-recognized by HLA-A^*^03:01, HLA-A^*^68:01, and HLA-A^*^11:01. Together with the current HLA-A^*^30:01, all of these HLA-A alleles are A3 supertype alleles (13, 19). At the same time, some peptides can also be cross-recognized by alleles from different HLA supertypes. We have also detected some influenza virus peptides that can be presented by both HLA-A11 and HLA-A24, which belong to different supertypes, A3 and A24, respectively (65). Herein, we found that the HIV peptide, RT313, could also be presented by A3 supertype allele HLA-A^*^30:01 and A1A3 supertype allele HLA-A^*^30:03. We also found that NP44, which was an A1 supertype allele, HLA-A^*^01:01-restricted peptide, could also be presented by HLA-A^*^30:03, but not by HLA-A^*^30:01 (17). This indicated that HLA-A^*^30:03 could cross-recognize peptides presented by A1A3 supertype alleles, but HLA-A^*^30:01 could only cross-recognize peptides presented by A3 supertype alleles. Previous work using recombinant MHC class I molecules, peptide MTB (QIMYNYPAM) has been shown to be presented by HLA-A^*^30:01 (18). Here, our results indicate that MTB bind to HLA-A^*^30:03, but not to HLA-A^*^30:01. The difference may be due to the different expression system in the studies.

In conclusion, we characterized the different peptide presentation features of two important HLA alleles, HLA-A^*^30:01 and HLA-A^*^30:03. Our results provide a better understanding of the molecular immunological characteristics of A1, A1A3, and A3 supertype molecules and also provide a beneficial reference for the screening of specific T-cell recognition peptides of viruses and tumors for the vaccine development or other immune interventions.

## Author Contributions

GG, WL, and YL conceptualized and designed the study. SZ, KL, YW, WX, HC, and YZ conducted the experiments. JL and CD provided experimental materials. YC and DL collected the data sets and solved the structures. WL, SZ, and KL analyzed the data and wrote the draft of the manuscript. GG and WL revised the manuscript. All authors contributed to manuscript revision, read and approved the submitted version.

### Conflict of Interest Statement

The authors declare that the research was conducted in the absence of any commercial or financial relationships that could be construed as a potential conflict of interest.

## Abbreviations

APCs: antigen-presenting cells
CD: circular dichroism
HLA: human leukocyte antigen
MHC: major histocompatibility complex
PBG: peptide-binding grooves
RMSD: root-mean-square deviation.

## References

[1] Sidney J del Guercio MF Southwood S Engelhard VH Appella E Rammensee HG. Several HLA alleles share overlapping peptide specificities. J Immunol. (1995) 154:247–59. 7527812

[2] del Guercio MF Sidney J Hermanson G Perez C Grey HM Kubo RT. Binding of a peptide antigen to multiple HLA alleles allows definition of an A2-like supertype. J Immunol. (1995) 154:685–93. 7529283

[3] Sidney J Grey HM Southwood S Celis E Wentworth PA del Guercio MF. Definition of an HLA-A3-like supermotif demonstrates the overlapping peptide-binding repertoires of common HLA molecules. Hum Immunol. (1996) 45:79–93. 8882405 10.1016/0198-8859(95)00173-5

[4] Sidney J Peters B Frahm N Brander C Sette A. HLA class I supertypes: a revised and updated classification. BMC Immunol. (2008) 9:1. 10.1186/1471-2172-9-1 18211710 PMC2245908

[5] Sette A Sidney J. Nine major HLA class I supertypes account for the vast preponderance of HLA-A and -B polymorphism. Immunogenetics. (1999) 50:201–12. 10602880 10.1007/s002510050594

[6] Lund O Nielsen M Kesmir C Petersen AG Lundegaard C Worning P. Definition of supertypes for HLA molecules using clustering of specificity matrices. Immunogenetics. (2004) 55:797–810. 10.1007/s00251-004-0647-4 14963618

[7] Lazaryan A Wang T Spellman SR Wang HL Pidala J Nishihori T. Human leukocyte antigen supertype matching after myeloablative hematopoietic cell transplantation with 7/8 matched unrelated donor allografts: a report from the Center for International Blood and Marrow Transplant Research. Haematologica. (2016) 101:1267–74. 10.3324/haematol.2016.143271 27247320 PMC5046657

[8] Ovsyannikova IG Jacobson RM Vierkant RA Pankratz VS Poland GA. HLA supertypes and immune responses to measles-mumps-rubella viral vaccine: findings and implications for vaccine design. Vaccine. (2007) 25:3090–100. 10.1016/j.vaccine.2007.01.020 17280755

[9] Dupont B. Nomenclature for factors of the HLA system, 1987. Decisions of the nomenclature committee on leukocyte antigens, which met in New York on November 21–23, 1987. Hum Immunol. (1989) 26:3–14. 10.1016/0198-8859(89)90027-X 2506155

[10] Krausa P Munz C Keilholz W Stevanovic S Jones EY Browning M. Definition of peptide binding motifs amongst the HLA-A^*^30 allelic group. Tissue Antigens. (2000) 56:10–8. 10.1034/j.1399-0039.2000.560102.x 10958351

[11] Bodmer JG Marsh SGE Albert ED Bodmer WF Dupont B Erlich HA. Nomenclature for factors of the HLA system, 1994. Tissue Antigens. (1994) 44:1–18. 7974464 10.1111/j.1399-0039.1994.tb02351.x

[12] Yang SY Population Analysis of Class I HLA Antigens by One- Dimensional Isoelectric Focusing Gel Electrophoresis: Workshop Summary Report. New York, NY: Springer (1989).

[13] Zhang S Liu J Cheng H Tan S Qi J Yan J. Structural basis of cross-allele presentation by HLA-A^*^0301 and HLA-A^*^1101 revealed by two HIV-derived peptide complexes. Mol Immunol. (2011) 49:395–401. 10.1016/j.molimm.2011.08.015 21943705

[14] Paximadis M Mathebula TY Gentle NL Vardas E Colvin M Gray CM. Human leukocyte antigen class I (A, B, C) and II (DRB1) diversity in the black and Caucasian South African population. Hum Immunol. (2012) 73:80–92. 10.1016/j.humimm.2011.10.013 22074999

[15] Nielsen M Lundegaard C Blicher T Lamberth K Harndahl M Justesen S. NetMHCpan, a method for quantitative predictions of peptide binding to any HLA-A and -B locus protein of known sequence. PLoS ONE. (2007) 2:e796. 10.1371/journal.pone.0000796 17726526 PMC1949492

[16] Lamberth K Roder G Harndahl M Nielsen M Lundegaard C Schafer-Nielsen C. The peptide-binding specificity of HLA-A^*^3001 demonstrates membership of the HLA-A3 supertype. Immunogenetics. (2008) 60:633–43. 10.1007/s00251-008-0317-z 18769915

[17] Quinones-Parra S Grant E Loh L Nguyen TH Campbell KA Tong SY. Preexisting CD8+ T-cell immunity to the H7N9 influenza A virus varies across ethnicities. Proc Natl Acad Sci USA. (2014) 111:1049–54. 10.1073/pnas.1322229111 24395804 PMC3903243

[18] Axelsson-Robertson R Ahmed RK Weichold FF Ehlers MM Kock MM Sizemore D. Human leukocyte antigens A^*^3001 and A^*^3002 show distinct peptide-binding patterns of the *Mycobacterium tuberculosis* protein TB10.4: consequences for immune recognition. Clin Vaccine Immunol. (2011) 18:125–134. 10.1128/CVI.00302-10 21084459 PMC3019778

[19] Niu L Cheng H Zhang S Tan S Zhang Y Qi J. Structural basis for the differential classification of HLA-A^*^6802 and HLA-A^*^6801 into the A2 and A3 supertypes. Mol Immunol. (2013) 55:381–92. 10.1016/j.molimm.2013.03.015 23566939 PMC7112617

[20] Hinrichs J Foll D Bade-Doding C Huyton T Blasczyk R Eiz-Vesper B. The nature of peptides presented by an HLA class I low expression allele. Haematologica. (2010) 95:1373–80. 10.3324/haematol.2009.016089 20220067 PMC2913087

[21] Gao GF Willcox BE Wyer JR Boulter JM O'Callaghan CA Maenaka K. Classical and nonclassical class I major histocompatibility complex molecules exhibit subtle conformational differences that affect binding to CD8alphaalpha. J Biol Chem. (2000) 275:15232–8. 10.1074/jbc.275.20.15232 10809759

[22] Garboczi DN Hung DT Wiley DC. HLA-A2-peptide complexes: refolding and crystallization of molecules expressed in *Escherichia coli* and complexed with single antigenic peptides. Proc Natl Acad Sci USA. (1992) 89:3429–33. 10.1073/pnas.89.8.3429 1565634 PMC48881

[23] Li X Liu J Qi J Gao F Li Q Li X. Two distinct conformations of a rinderpest virus epitope presented by bovine major histocompatibility complex class I N^*^01801: a host strategy to present featured peptides. J Virol. (2011) 85:6038–48. 10.1128/JVI.00030-11 21450819 PMC3126294

[24] Li H Zhou M Han J Zhu X Dong T Gao GF. Generation of murine CTL by a hepatitis B virus-specific peptide and evaluation of the adjuvant effect of heat shock protein glycoprotein 96 and its terminal fragments. J Immunol. (2005) 174:195–204. 10.4049/jimmunol.174.1.195 15611241

[25] Liu WJ Lan J Liu K Deng Y Yao Y Wu S. Protective T Cell Responses Featured by Concordant Recognition of Middle East Respiratory Syndrome Coronavirus-Derived CD8+ T Cell Epitopes and Host MHC. J Immunol. (2017) 198:873–82. 10.4049/jimmunol.1601542 27903740

[26] Otwinowski Z Minor W Processing of X-ray diffraction data collected in oscillation mode. Methods Enzymol. (1997) 276:307–26. 10.1016/S0076-6879(97)76066-X 27754618

[27] Read RJ. Pushing the boundaries of molecular replacement with maximum likelihood. Acta Crystallogr D Biol Crystallogr. (2001) 57(Pt 10):1373–82. 10.1107/s0907444901012471 11567148

[28] Collaborative Computational Project Number The CCP4 suite: programs for protein crystallography. Acta Crystallogr D Biol Crystallogr. (1994) 50(Pt 5):760–3. 10.1107/S0907444994003112 15299374

[29] Emsley P Cowtan K. Coot: model-building tools for molecular graphics. Acta Crystallogr D Biol Crystallogr. (2004) 60(Pt 12):2126–32. 10.1107/S090744490401(9158) 15572765

[30] Murshudov GN Vagin AA Dodson EJ. Refinement of macromolecular structures by the maximum-likelihood method. Acta Crystallogr D Biol Crystallogr. (1997) 53(Pt 3):240–55. 10.1107/S0907444996012255 15299926

[31] Adams PD Afonine PV Bunkoczi G Chen VB Davis IW Echols N. PHENIX: a comprehensive Python-based system for macromolecular structure solution. Acta Crystallogr D Biol Crystallogr. (2010) 66(Pt 2):213–21. 10.1107/S090744490905(2925) 20124702 PMC2815670

[32] Liu WJ Tan S Zhao M Quan C Bi Y Wu Y. Cross-immunity against avian influenza A(H7N9) virus in the healthy population is affected by antigenicity-dependent substitutions. J Infect Dis. (2016) 214:1937–46. 10.1093/infdis/jiw471 27738054

[33] Thompson JD Gibson TJ Plewniak F Jeanmougin F Higgins DG. The CLUSTAL_X windows interface: flexible strategies for multiple sequence alignment aided by quality analysis tools. Nucleic Acids Res. (1997) 25:4876–4882. 9396791 10.1093/nar/25.24.4876 PMC147148

[34] Gouet P Robert X Courcelle E. ESPript/ENDscript: Extracting and rendering sequence and 3D information from atomic structures of proteins. Nucleic Acids Res. (2003) 31:3320–3. 10.1093/nar/gkg556 12824317 PMC168963

[35] Rudolph MG Stanfield RL Wilson IA. How TCRs bind MHCs, peptides, and coreceptors? Annu Rev Immunol. (2006) 24:419–66. 10.1146/annurev.immunol.23.021704.115658 16551255

[36] Stewart-Jones GB McMichael AJ Bell JI Stuart DI Jones EY A structural basis for immunodominant human T cell receptor recognition. Nat Immunol. (2003) 4:657–63. 10.1038/ni942 12796775

[37] Shi Y Kawana-Tachikawa A Gao F Qi J Liu C Gao J Conserved Vdelta1 binding geometry in a setting of locus-disparate pHLA recognition by delta/alphabetaTCRs: insight into recognition of HIV peptides by TCR. J Virol. (2017) 91:e00725–17. 10.1128/JVI.00725-17 28615212 PMC5553175

[38] Liang R Sun Y Liu Y Wang J Wu Y Li Z. Major histocompatibility complex class I (FLA-E^*^01801) molecular structure in domestic cats demonstrates species-specific characteristics in presenting viral antigen peptides. J Virol. (2018) 92. 10.1128/JVI.01631-17 29263258 PMC5827386

[39] Xiao J Xiang W Chai Y Haywood J Qi J Ba L. Diversified Anchoring Features the Peptide Presentation of DLA-88^*^50801: First Structural Insight into Domestic Dog MHC Class I. J Immunol. (2016) 197:2306–15. 10.4049/jimmunol.1600887 27511732

[40] Speir JA Stevens J Joly E Butcher GW Wilson IA. Two different, highly exposed, bulged structures for an unusually long peptide bound to rat MHC class I RT1-Aa. Immunity. (2001) 14:81–92. 10.1016/s1074-7613(01)00091-7 11163232

[41] Chappell P Meziane el K Harrison M Magiera L Hermann C Mears L. Expression levels of MHC class I molecules are inversely correlated with promiscuity of peptide binding. Elife. (2015) 4:e0. 10.7554/eLife.0(5345) 25860507 PMC4420994

[42] Hundal J Kiwala S Feng YY Liu CJ Govindan R Chapman WC. Accounting for proximal variants improves neoantigen prediction. Nat Genet. (2019) 51:175–9. 10.1038/s41588-018-0283-9 30510237 PMC6309579

[43] Andreatta M Nielsen M. Gapped sequence alignment using artificial neural networks: application to the MHC class I system. Bioinformatics. (2016) 32:511–7. 10.1093/bioinformatics/btv639 26515819 PMC6402319

[44] Nielsen M Lundegaard C Worning P Lauemoller SL Lamberth K Buus S. Reliable prediction of T-cell epitopes using neural networks with novel sequence representations. Protein Sci. (2003) 12:1007–17. 10.1110/ps.0239403 12717023 PMC2323871

[45] Ott PA Hu Z Keskin DB Shukla SA Sun J Bozym DJ. An immunogenic personal neoantigen vaccine for patients with melanoma. Nature. (2017) 547:217–21. 10.1038/nature22991 28678778 PMC5577644

[46] Li F Chen C Ju T Gao J Yan J Wang P. Rapid tumor regression in an Asian lung cancer patient following personalized neo-epitope peptide vaccination. Oncoimmunology. (2016) 5:e1238539. 10.1080/2162402X.2016.1238539 28123873 PMC5214696

[47] Zhou M Xu D Li X Li H Shan M Tang J. Screening and identification of severe acute respiratory syndrome-associated coronavirus-specific CTL epitopes. J Immunol. (2006) 177:2138–45. 10.4049/jimmunol.177.4.2138 16887973

[48] Xu K Song Y Dai L Zhang Y Lu X Xie Y. Recombinant chimpanzee adenovirus vaccine AdC7-M/E protects against zika virus infection and testis damage. J Virol. (2018) 92:e01722–17. 10.1128/JVI.01722-17 29298885 PMC5827382

[49] Zhao M Liu K Luo J Tan S Quan C Zhang S. Heterosubtypic protections against human-infecting avian influenza viruses correlate to biased cross-T-cell responses. MBio. (2018) 9:e01408–18. 10.1128/mBio.01408-18 30087171 PMC6083907

[50] Sahin U Derhovanessian E Miller M Kloke BP Simon P Lower M. Personalized RNA mutanome vaccines mobilize poly-specific therapeutic immunity against cancer. Nature. (2017) 547:222–6. 10.1038/nature23003 28678784

[51] Zhao M Chen J Tan S Dong T Jiang H Zheng J. Prolonged evolution of virus-specific memory T cell immunity after severe avian Influenza A (H7N9) virus infection. J Virol. (2018) 92:e01024–18. 10.1128/JVI.01024-18 29925664 PMC6096810

[52] Schumacher TN Schreiber RD. Neoantigens in cancer immunotherapy. Science. (2015) 348:69–74. 10.1126/science.aaa4971 25838375

[53] Schumacher TN Scheper W Kvistborg P. Cancer Neoantigens. Annu Rev Immunol. (2019) 37:173–200. 10.1146/annurev-immunol-042617-053402 30550719

[54] Corbett AJ Eckle SB Birkinshaw RW Liu L Patel O Mahony J. T-cell activation by transitory neo-antigens derived from distinct microbial pathways. Nature. (2014) 509:361–5. 10.1038/nature13160 24695216

[55] Yadav M Jhunjhunwala S Phung QT Lupardus P Tanguay J Bumbaca S. Predicting immunogenic tumour mutations by combining mass spectrometry and exome sequencing. Nature. (2014) 515:572–6. 10.1038/nature14001 25428506

[56] Hinrichs CS Rosenberg SA. Exploiting the curative potential of adoptive T-cell therapy for cancer. Immunol Rev. (2014) 257:56–71. 10.1111/imr.12132 24329789 PMC3920180

[57] Zacharakis N Chinnasamy H Black M Xu H Lu YC Zheng Z. Immune recognition of somatic mutations leading to complete durable regression in metastatic breast cancer. Nat Med. (2018) 24:724–30. 10.1038/s41591-018-0040-8 29867227 PMC6348479

[58] Lu YC Yao X Crystal JS Li YF El-Gamil M Gross C. Efficient identification of mutated cancer antigens recognized by T cells associated with durable tumor regressions. Clin Cancer Res. (2014) 20:3401–10. 10.1158/1078-0432.CCR-14-0433 24987109 PMC4083471

[59] Butter C Staines K van Hateren A Davison TF Kaufman J. The peptide motif of the single dominantly expressed class I molecule of the chicken MHC can explain the response to a molecular defined vaccine of infectious bursal disease virus (IBDV). Immunogenetics. (2013) 65:609–18. 10.1007/s00251-013-0705-x 23644721 PMC3710569

[60] Hofmann A Plachy J Hunt L Kaufman J Hala K. v-src oncogene-specific carboxy-terminal peptide is immunoprotective against Rous sarcoma growth in chickens with MHC class I allele B-F12. Vaccine. (2003) 21:4694–9. 10.1016/S0264-410X(03)00516-4 14585677

[61] Wang HY Cui Z Xie LJ Zhang LJ Pei ZY Chen FJ. HLA class II alleles differing by a single amino acid associate with clinical phenotype and outcome in patients with primary membranous nephropathy. Kidney Int. (2018) 94:974–82. 10.1016/j.kint.2018.06.005 30173899

[62] Zhao M Zhang H Liu K Gao GF Liu WJ. Human T-cell immunity against the emerging and re-emerging viruses. Sci China Life Sci. (2017) 60:1307–16. 10.1007/s11427-017-9241-3 29294219 PMC7089170

[63] Liu WJ Zhao M Liu K Xu K Wong G Tan W. T-cell immunity of SARS-CoV: Implications for vaccine development against MERS-CoV. Antiviral Res. (2017) 137:82–92. 10.1016/j.antiviral.2016.11.006 27840203 PMC7113894

[64] Wen J Tang WW Sheets N Ellison J Sette A Kim K. Identification of Zika virus epitopes reveals immunodominant and protective roles for dengue virus cross-reactive CD8(+) T cells. Nat Microbiol. (2017) 2:17036. 10.1038/nmicrobiol.2017.36 28288094 PMC5918137

[65] Liu J Zhang S Tan S Yi Y Wu B Cao B. Cross-allele cytotoxic T lymphocyte responses against 2009 pandemic H1N1 influenza A virus among HLA-A24 and HLA-A3 supertype-positive individuals. J Virol. (2012) 86:13281–94. 10.1128/JVI.01841-12 23015716 PMC3503122

