# Supplementary material for: Divergent Peptide Presentations of HLA-A*30 Alleles Revealed by Structures With Pathogen Peptides
Source: Front Immunol. 2019 Jul 23;10:1709. doi: 10.3389/fimmu.2019.01709 (PMC6664060; doi:10.3389/fimmu.2019.01709)
Supplement: Supplementary file 2 [file Data_Sheet_1.PDF]

Supplemental Figures and Tables

Supplemental Figure 1

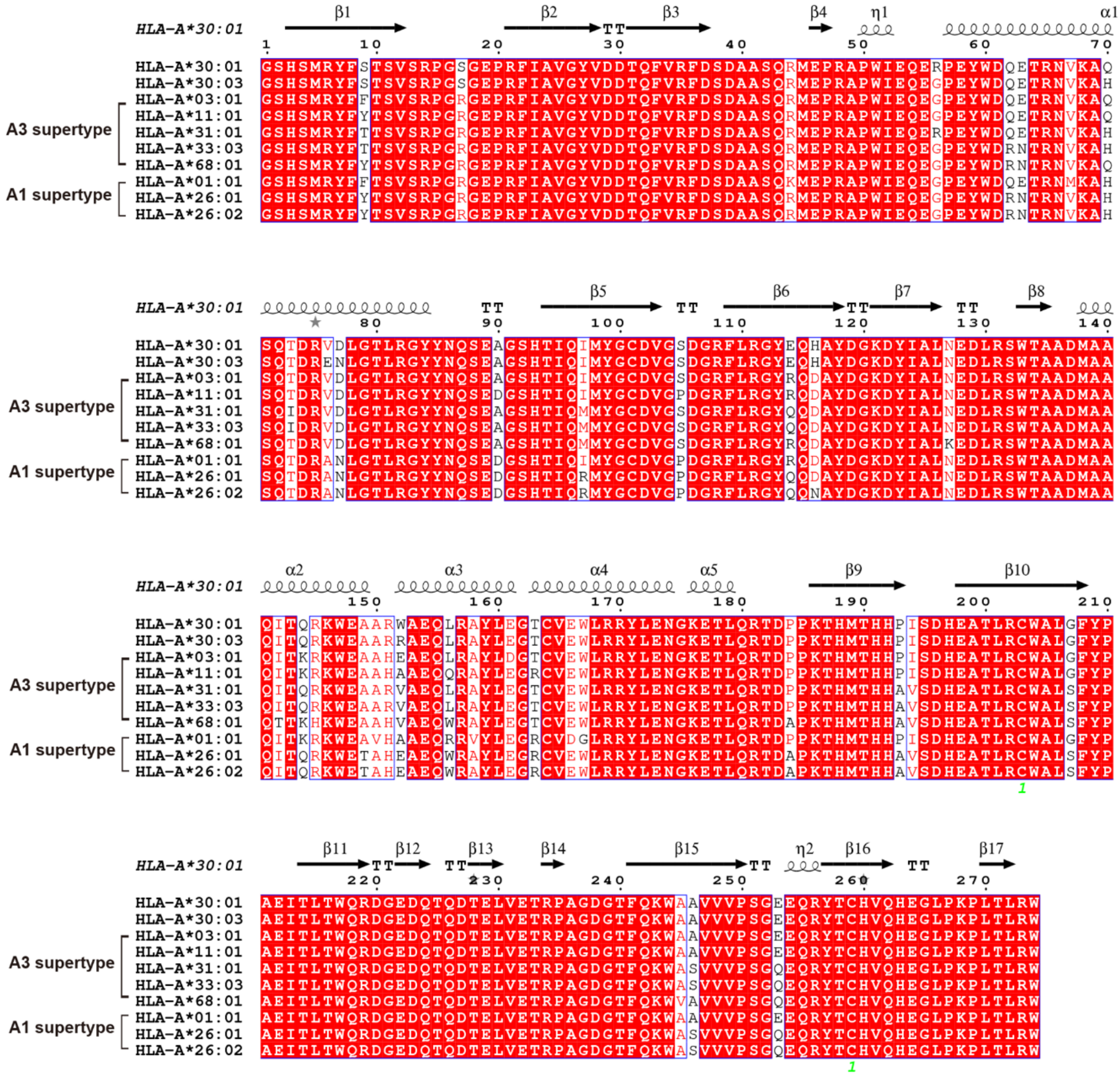

Supplemental Figure 1. Structure-based sequence alignment of some alleles of HLA-A1 and HLA-A3 Supertypes. Cylinders indicate α-helices, and black arrows indicate β-strands. Amino acids highlighted in red are completely conserved and those in blue boxes are highly (> 80%) conserved.

Supplemental Figure 2

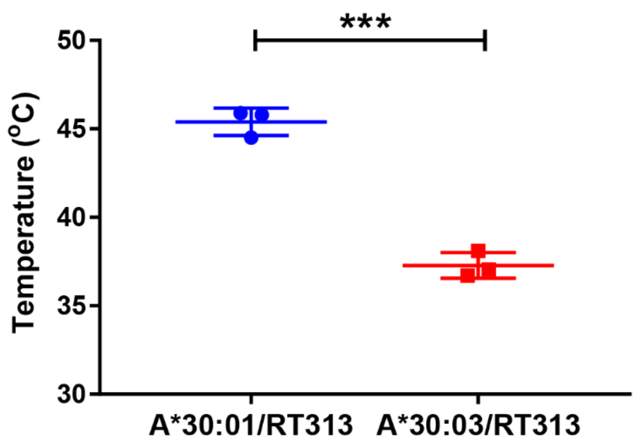

Supplemental Figure 2. Thermostabilities of HLA-A*30:01 and HLA-A*30:03 complexed with RT313. The thermostabilities of HLA-A*30:01 and HLA-A*30:03 complexed with RT313 were tested by circular dichromism (CD) spectroscopy. The temperature was increased by 1°C/min. The curves for the unfolded fractions were determined by monitoring the CD value at 218 nm. Each complex was tested three times. Statistical significance of the melting temperatures of RT313 presented by HLA-A*30:01 and HLA-A*30:03 were analysed by Student's t-test: *P < 0.05, **P < 0.01 and ****P < 0.0001.

Supplemental Figure 3

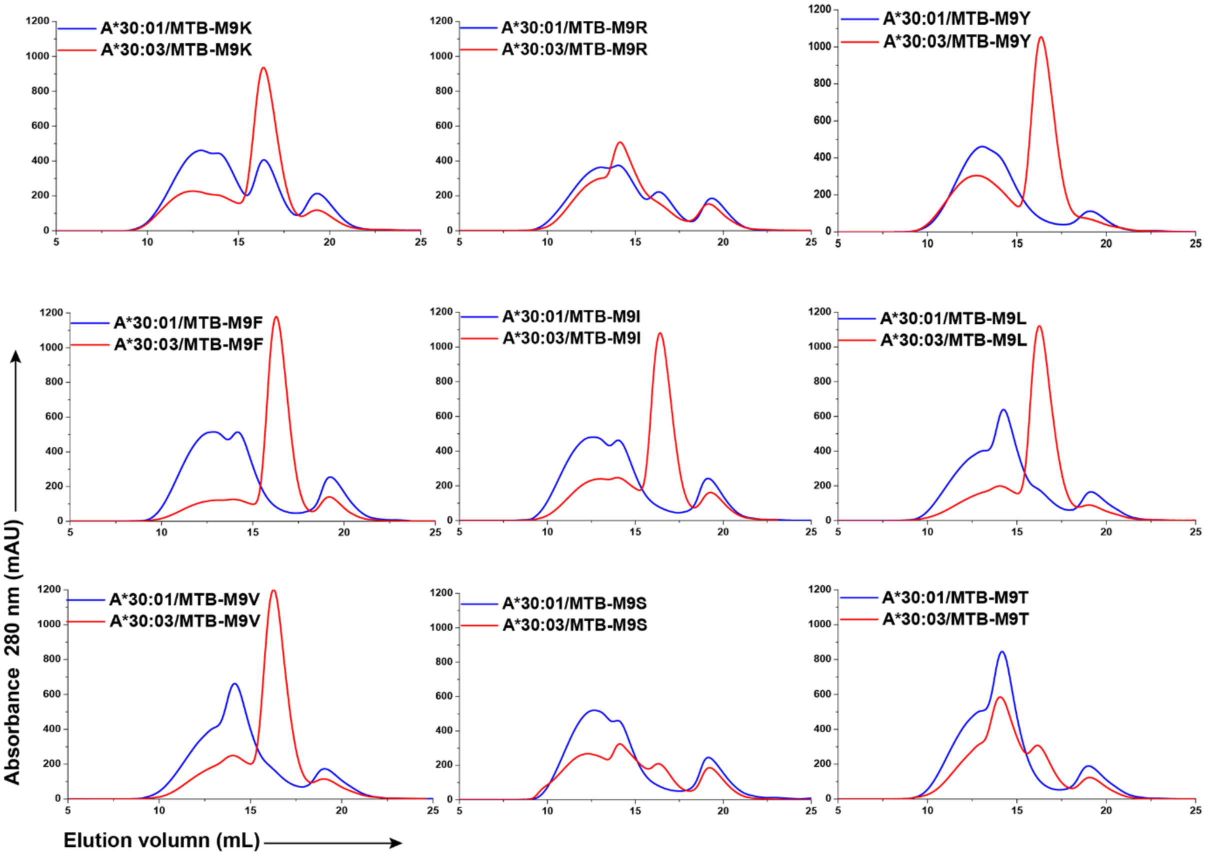

Supplemental Figure 3. The binding capabilities of MTB PΩ-residue mutated peptides with HLA-A*30:01 or HLA-A*30:03. MTB PΩ-residue mutated peptides (MTB-M9K, MTB-M9R, MTB-M9Y, MTB-M9F, MTB-M9I, MTB-M9L, MTB-M9V, MTB-M9S, MTB-M9T) presented by HLA-A*30:01 (blue) or HLA-A*30:03 (red) were elucidated by *in vitro* refolding assays. The high absorbance peaks of the HLAs with the expected molecular mass of 45 KD were eluted at an estimated volume of 16 mL on a Superdex^{TM} 200 10/300 GL column.

Supplemental Figure 4

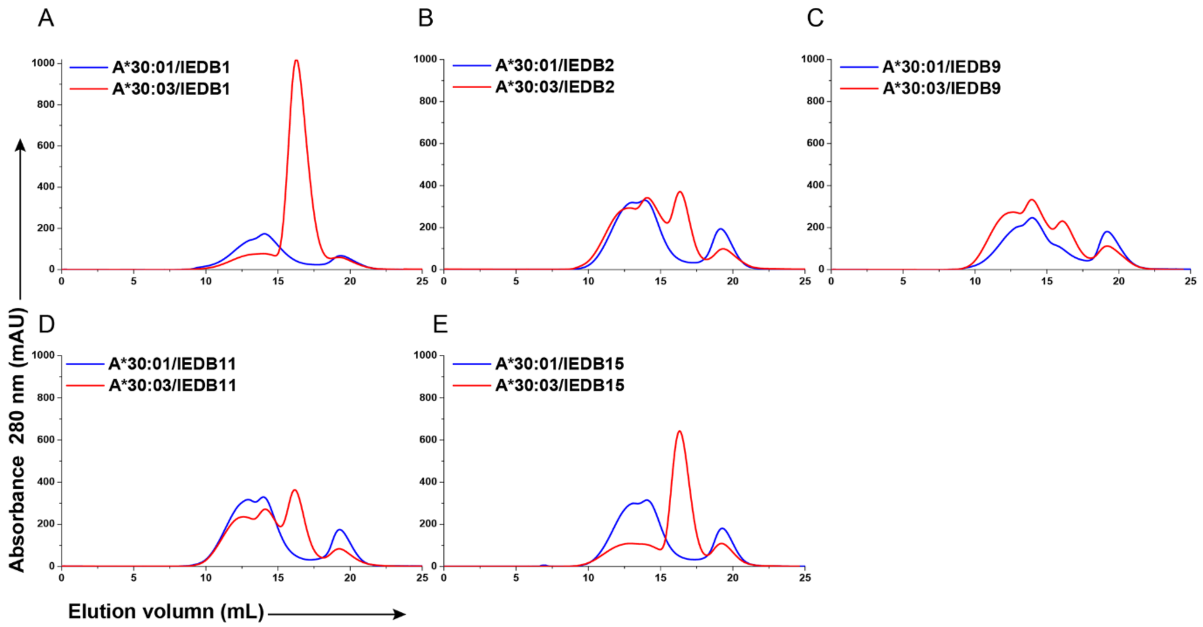

Supplemental Figure 4. The binding capabilities of peptides with HLA-A*30:01 and HLA-A*30:03. Peptides were retrieved from the IEDB (http://www.iedb.org) **(A**: IEDB1, **B**: IEDB2, **C**: IEDB9, **D**: IEDB11, and **E**: IEDB15**)** and the binding by HLA-A*30:01 (blue) or HLA-A*30:03 (red) were elucidated by *in vitro* refolding assays. The high absorbance peaks of the HLAs with the expected molecular mass of 45 KD were eluted at an estimated volume of 16 mL on a Superdex^{TM} 200 10/300 GL column.

Supplemental Figure 5

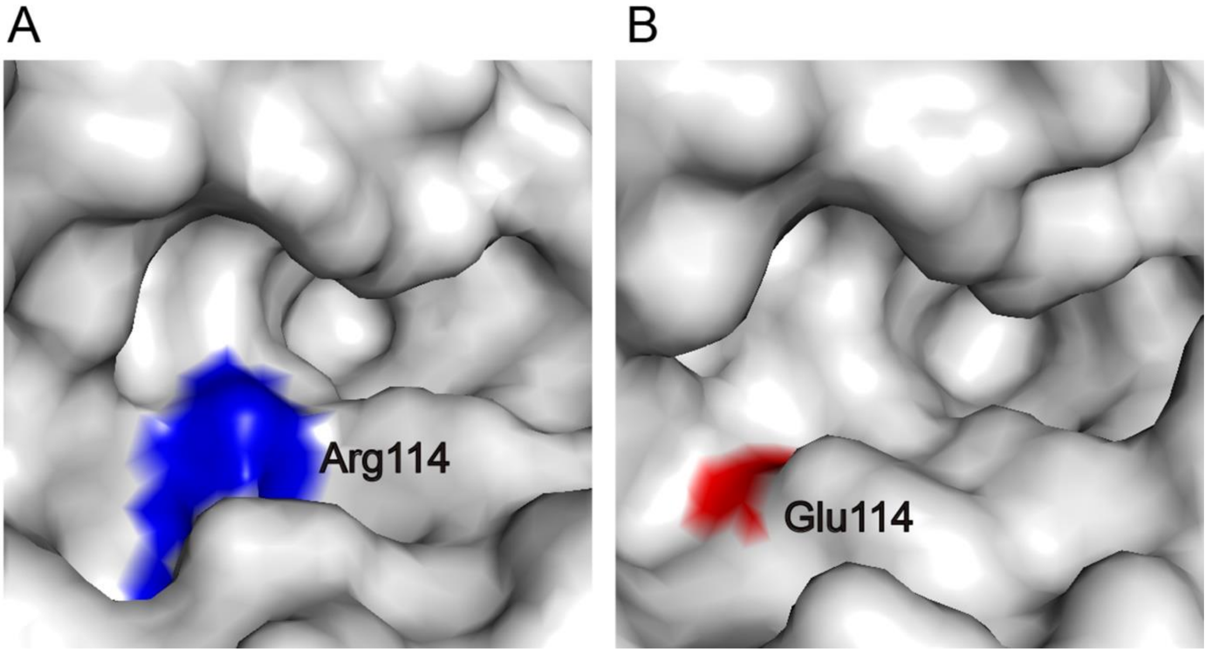

Supplemental Figure 5. Different locations of residue 114 in HLA-A*01:01 and HLA-A*30:03. (A) Arg114 (blue) in HLA-A*01:01 participates in the composition of the F pocket. **(B)** Glu114 (red) in HLA-A*30:03 was far away from F pocket.

Supplemental Figure 6

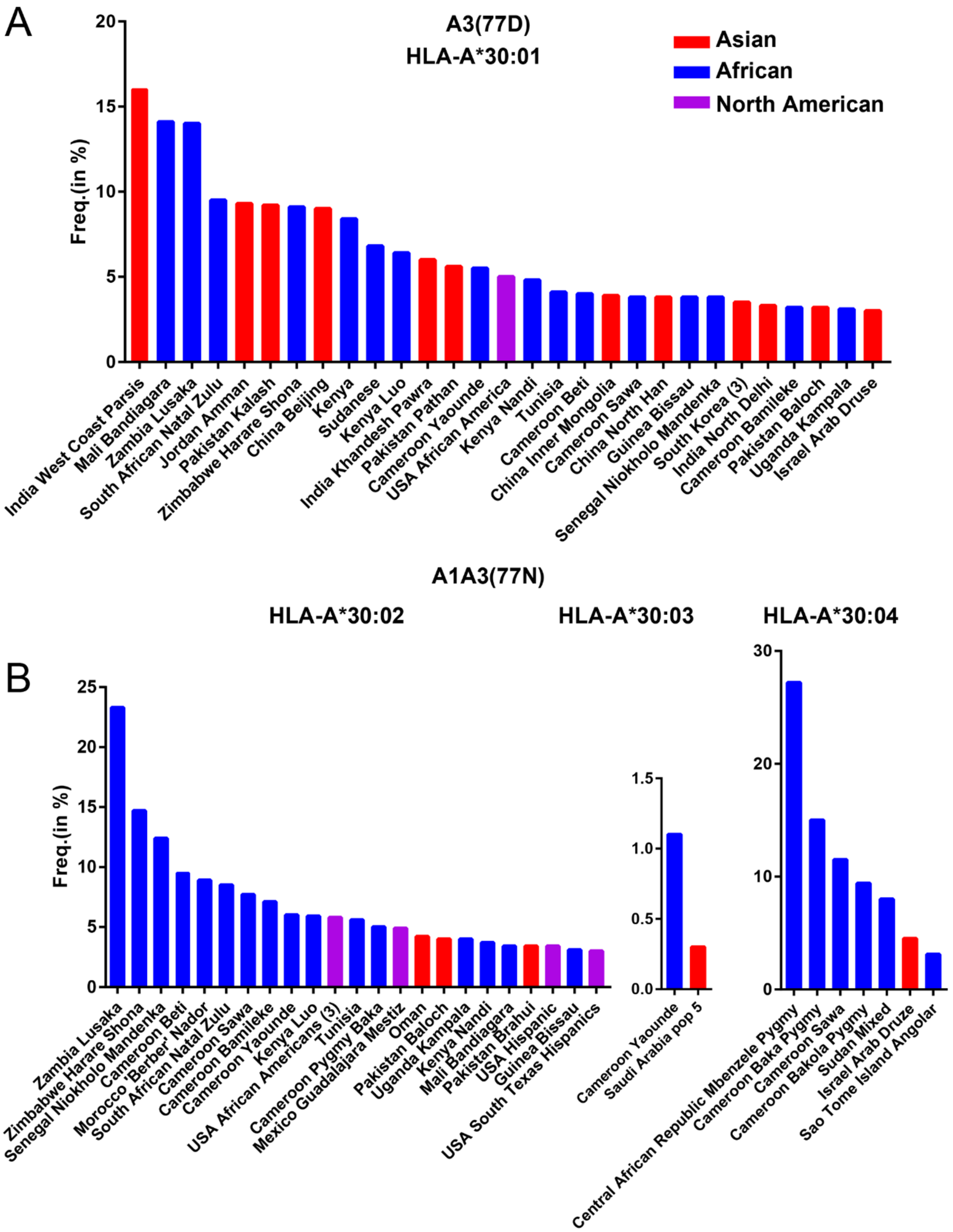

Supplemental Figure 6. The frequency of HLA-A*30:01, HLA-A*30:02, HLA-A*30:03, and HLA-A*30:04 in different regions. (A) The HLA-A*30:01-carrying population mainly distribute in Asia(red), Africa (blue), and North American(purple). The frequencies of HLA-A*30:01 in India West Coast Parsis is up to 16%. **(B)** The distribution of HLA-A*30:02, HLA-A*30:03, and HLA-A*30:04-carrying population. HLA-A*30:02 and

HLA-A*30:04-carrying population is mainly located in Africa. The highest percentage of HLA-A*30:02 is Lusaka, Zambia which could up to 23.3%. HLA-A*30:04 in the Mbenzele Pygmies from the Central African Republic is up to 27.2%. HLA-A*30:03 is mainly located in Yaounde, Cameroon with a frequency of 1.1%.

Supplemental Table 2. Two groups of A*30 serotypes based on structurally-determined key residues

		F pocket			
		74	77	114	116
A3 supertype	HLA-A*30:01[a]	**D**	**D**	**E**	**H**
	HLA-A*30:08	**D**	**D**	**E**	**H**
	HLA-A*30:11	**D**	**D**	**E**	**H**
	HLA-A*30:14L	**D**	**D**	**E**	**H**
	HLA-A*30:15	**D**	**D**	**E**	**H**
	HLA-A*30:17	**D**	**D**	**E**	**H**
	HLA-A*30:18	**D**	**D**	**E**	**H**
	HLA-A*30:19	**D**	**D**	**E**	**H**
	HLA-A*30:23	**D**	**D**	**E**	**H**
	HLA-A*30:24	**D**	**D**	**E**	**H**
A1A3 supertype	HLA-A*30:03	**D**	**N**	**E**	**H**
	HLA-A*30:02	**D**	**N**	**E**	**H**
	HLA-A*30:04	**D**	**N**	**E**	**H**
	HLA-A*30:06	**D**	**N**	**E**	**H**
	HLA-A*30:07	**D**	**N**	**E**	**H**
	HLA-A*30:09	**D**	**N**	**E**	**H**
	HLA-A*30:10	**D**	**N**	**E**	**H**
	HLA-A*30:12	**D**	**N**	**E**	**H**
	HLA-A*30:22	**D**	**N**	**E**	**H**
	HLA-A*30:25	**D**	**N**	**E**	**H**

[a]Protein sequences of A*30 serotypes were obtained from https://www.ebi.ac.uk/ipd/imgt/hla.
